# *Anopheles stephensi* Feeding, Flight Behavior, and Infection With Malaria Parasites are Altered by Ingestion of Serotonin

**DOI:** 10.3389/fphys.2022.911097

**Published:** 2022-06-07

**Authors:** Anna M. Briggs, Malayna G. Hambly, Raquel M. Simão-Gurge, Sarah M. Garrison, Zainab Khaku, Grace Van Susteren, Edwin E. Lewis, Jeffrey A. Riffell, Shirley Luckhart

**Affiliations:** ^1^ Department of Entomology, Plant Pathology and Nematology, University of Idaho, Moscow, ID, United States; ^2^ Department of Biology, University of Washington, Seattle, WA, United States; ^3^ Department of Biological Sciences, University of Idaho, Moscow, ID, United States

**Keywords:** 5-hydroxytryptamine, serotonin, *Plasmodium yoelii*, *Plasmodium falciparum*, *Anopheles stephensi*, flight behavior, lifespan, fecundity

## Abstract

Approximately 3.4 billion people are at risk of malaria, a disease caused by infection with *Plasmodium* spp. parasites, which are transmitted by *Anopheles* mosquitoes. Individuals with severe falciparum malaria often exhibit changes in circulating blood levels of biogenic amines, including reduced serotonin or 5-hydroxytryptamine (5-HT), and these changes are associated with disease pathology. In insects, 5-HT functions as an important neurotransmitter for many behaviors and biological functions. In *Anopheles stephensi*, we show that 5-HT is localized to innervation in the head, thorax, and midgut, suggesting a gut-to-brain signaling axis that could support the effects of ingested 5-HT on mosquito biology and behavioral responses. Given the changes in blood levels of 5-HT associated with severe malaria and the key roles that 5-HT plays in insect neurophysiology, we investigated the impact of ingesting blood with healthy levels of 5-HT (1.5 µM) or malaria-associated levels of 5-HT (0.15 µM) on various aspects of *A. stephensi* biology. In these studies, we provisioned 5-HT and monitored fecundity, lifespan, flight behavior, and blood feeding of *A. stephensi.* We also assessed the impact of 5-HT ingestion on infection of *A. stephensi* with the mouse malaria parasite *Plasmodium yoelii yoelii* 17XNL and the human malaria parasite *Plasmodium falciparum*. Our data show that ingestion of 5-HT associated with severe malaria increased mosquito flight velocity and investigation of visual objects in response to host odor (CO_2_). 5-HT ingestion in blood at levels associated with severe malaria also increased the tendency to take a second blood meal 4 days later in uninfected *A. stephensi*. In mosquitoes infected with *P. y. yoelii* 17XNL, feeding tendency was decreased when midgut oocysts were present but increased when sporozoites were present. In addition to these effects, treatment of *A. stephensi* with 5-HT associated with severe malaria increased infection success with *P. y. yoelii* 17XNL compared to control, while treatment with healthy levels of 5-HT decreased infection success with *P. falciparum.* These changes in mosquito behavior and infection success could be used as a basis to manipulate 5-HT signaling in vector mosquitoes for improved control of malaria parasite transmission.

## 1 Introduction

Infection with *Plasmodium* spp. parasites, the causative agents of malaria transmitted by *Anopheles* mosquitoes, threatens approximately 3.4 billion people, with the largest proportion of deaths caused by infection with *Plasmodium falciparum* (World Health Organization, 2020). Individuals with severe falciparum malaria exhibit changes in circulating blood levels of biogenic amines, including reduced serotonin or 5-hydroxytryptamine (5-HT), and these alterations have been associated with disease pathology (Enwonwu et al., 1999; Lopansri et al., 2006). Low 5-HT levels can result from reduced levels of the 5-HT precursor tryptophan, an outcome associated with hyperphenylalaninemia, or from alternative metabolism of tryptophan or elevated turnover of 5-HT (Enwonwu et al., 1999; Dobbie et al., 2000; Nishanth and Schlüter, 2019). In normal healthy adults, blood 5-HT ranges from 0.6 µM to 1.5 µM, and in normal healthy children, blood 5-HT ranges from 0.4 µM to 2.6 µM (Loo, 1974; Badcock et al., 1987; Winn et al., 2018). Severe malaria has been associated with 5-HT levels as low as 0.1 µM; low blood 5-HT is reported to be associated with high falciparum gametocyte conversion rates and high blood 5-HT is associated with low falciparum gametocyte conversion rates (Usui et al., 2019). These observations suggest that mosquitoes could ingest transmissible gametocytes with blood 5-HT concentrations that vary as much as 10-fold depending on disease severity.

5-HT is an insect neuromodulator that regulates life history traits and behavior, based on studies of endogenous 5-HT signaling and treatment with 5-HT. Ingestion of 5-HT by *Drosophila melanogaster* resulted in significantly decreased oviposition success compared to control insects (Thomas et al., 1998). Disruption of the 5-HT pathway in *D. melanogaster* has also been observed to alter feeding choices and lifespan (Ro et al., 2016), with 5-HT receptor function linked to learning, gustation, flight, and other behaviors (Johnson et al., 2011; Ro et al., 2016; Huser et al., 2017). In *Aedes aegypti*, reduced 5-HT levels were associated with an increased tendency to blood feed and increased flight distance (Ngai et al., 2019), however in *Aedes triseriatus*, depletion of 5-HT levels was associated with reduced blood feeding success relative to controls (Novak and Rowley, 1994) indicating there are some discrepancies on the impact of 5-HT on feeding. In other studies, Ling and Raikhel (2018) reported effects of gene-editing disruption of serotonergic signaling on insulin-like peptide-mediated control of *A. aegypti* metabolism, reproduction, and body size.

Given this context, we investigated the impact of ingested 5-HT on *Anopheles stephensi*, the highly invasive Indian malaria mosquito (Sinka et al., 2020). In these studies, we provisioned 5-HT at normal blood levels (1.5 µM) and levels associated with severe malaria (0.15 µM) and monitored adult female mosquito fecundity, flight behavior, lifespan, and blood-feeding behavior. We also assessed the impact of 5-HT ingestion at these levels on *A. stephensi* infection with the mouse malaria parasite *Plasmodium yoelii yoelii* 17XNL and with *P. falciparum*. Our data show that 5-HT ingested at severe malaria levels altered *A. stephensi* feeding and flight behaviors, and tendency to take a subsequent blood meal at critical timepoints in parasite development following 5-HT treatment. Ingestion of 5-HT at reduced levels associated with severe malaria impacted infection success and feeding behavior of infected mosquitoes. Taken together, these data suggest that reduced 5-HT levels associated with severe malaria would favor malaria parasite transmission, supporting the potential for novel mosquito-targeted interventions for malaria control.

## 2 Materials and Methods

### 2.1 Materials

5-HT hydrochloride, 98% (Alfa Aesar, Haverhill, MA); human red blood cells (RBCs, Interstate Blood Bank, Memphis, TN, United States); human serum (Interstate Blood Bank, Memphis, TN, United States); RPMI-1640 medium (Gibco, Gaithersburg, MD, United States) supplemented with HEPES, L-glutamine, hypoxanthine (Acros Organics), DL-lactic acid (Acros Organics); Hoechst 33342-trihydrochloride trihydrate (Invitrogen, Carlsbad, CA, United States); JC-1 (Invitrogen, Carlsbad, CA, United States); mercurochrome, Ringer’s solution (3 mM CaCl_2_, 182 mM KCl, 46 mM NaCl, 10 mM Tris pH 7.2), 1-octen-3-ol (Sigma-Aldrich); electrode gel (Parker Laboratories, Fairfield, NJ, United States); N-3-dimethylaminopropyl-N′-ethylcarbodiimide (Sigma-Aldrich, St. Louis, MO, United States, cat. #03449); paraformaldehyde (Electron Microscopy Sciences, Hatfield, PA, United States, cat. #15710); and agarose (Sigma-Aldrich) were purchased for these studies. The following reagents were purchased for immunohistochemistry: goat serum (BSA; Jackson ImmunoResearch Laboratories, West Grove, PA, United States, cat. #001-000-162); rabbit anti-histamine (ImmunoStar, Hudson, WI, United States, cat. #22939, RRID:AB_572245); goat anti-5-HT (ImmunoStar, Hudson, WI, United States, cat. #20079); Alexa Fluor 488 (Thermo Fisher, cat. #A-11008); Cy5 (Abcam, Cambridge, MA 02139, #ab6566); and Vectashield®PLUS (Vector Laboratories, Burlingame, CA, cat. #H-1900).

### 2.2 Mosquito Rearing

Mosquitoes used in these experiments were derived from a colony of Indian wild type strain of *A. stephensi* Liston from the Luckhart lab colony, maintained since 1998. This colony was initiated from a colony maintained by the Department of Entomology at Walter Reed Army Institute of Research (WRAIR, Washington, DC). Mosquito rearing and maintenance were performed as previously described (Rodriguez et al., 2021). Mosquitoes at all life stages and during experiments were kept at 27°C with a 12 h light-dark cycle (0800-2000) and adults were maintained at 80% humidity. Mosquitoes used in these studies were collected, at minimum, 24 h prior to the start of an experiment, were 4–8 days old at the start of all experiments and were housed in 2 L polypropylene containers with mesh screening.

### 2.3 Histamine/5-HT Immunohistochemistry

For immunohistochemical staining of histaminergic tissues, *A. stephensi* head, thorax, and abdomen samples were dissected and fixed in 4% N-3-dimethylaminopropyl-N′-ethylcarbodiimide in 0.01 M phosphate-buffered saline (PBS) at 4°C for 4 h. A similar protocol was used for 5-HT staining, except that the 4% N-3-dimethylaminopropyl-N′-ethylcarbodiimide was not used. Tissues were then transferred and fixed in 4% paraformaldehyde in PBS overnight at 4°C. Following fixation, tissues were washed in PBS twice and embedded in 7% agarose and sectioned at 60 μm using a Leica VT 1200S vibratome. Tissues were washed in PBS with 0.5% TritonX (PBT) and blocked in 5% goat serum for 1 h. Tissues were placed on a shaker and then incubated in 1:500 rabbit anti-histamine and 1:250 anti-5-HT with 5% goat serum in PBT overnight at room temperature. After primary antibody incubation, tissues were washed six times in PBT, blocked in goat serum, and incubated in 1:1000 Alexa Fluor 488 and 1:100 Cy5. Tissues were then washed in PBT and PBS, processed through an ascending glycerol gradient and mounted in Vectashield®PLUS. These histamine and 5-HT antibodies have shown to be specific and effective in a variety of invertebrate species, including *D. melanogaster* (Bradley et al., 2016; Chapman et al., 2017; Chapman et al., 2018). Preabsorption controls with histamine and 5-HT eliminated any labeling.

### 2.4 Priming *A. stephensi* With 5-HT

Testing the effects of 5-HT on infection of *A. stephensi* with *P. y. yoelii* required delivery by priming to mosquitoes prior to feeding on infected mice and was performed as described previously (Rodriguez et al., 2021). Priming was also used for a subset of behavioral studies with uninfected *A. stephensi* for a direct comparison with *P. y. yoelii* infection studies. For priming, 80-120 female mosquitoes housed in 2 L polypropylene containers with a mesh screen received soaked cotton balls and a sugar cube. Control mosquitoes received cotton balls soaked with water only, while treated mosquitoes received soaked cotton balls with severe malaria-associated 0.15 µM 5-HT in water, or soaked cotton balls with 1.5 µM 5-HT in water to represent healthy levels of 5-HT. It is important to note that mosquitoes would not ingest blood from a vertebrate host with no 5-HT present, but that these controls represent standard feeding conditions in the lab. Treatment solutions were made fresh daily, and cotton balls for control and treatment groups were changed twice per day, between 0830-1000 and 1630-1800, for 3 days prior to the first bloodmeal. Within 30 min to 1 h before the blood meal, the soaked cotton balls and sugar cube were removed from the 2 L containers.

### 2.5 Delivery of an Artificial Blood Meal to *A. stephensi*

Glass bell feeders were used to deliver a blood meal consisting of washed human RBCs and heat inactivated human serum (1:1, vol:vol) to mosquitoes for 15 min. Unless otherwise stated, blood meals were offered between 0800-1100. This timing was maintained for consistency across studies. Although time of feeding has been shown to alter some reproductive variables, it does not impact infection success (O’Donnell et al., 2019). Female mosquitoes were maintained on 10% sucrose-soaked cotton balls between blood meals.

### 2.6 Provisioning of 5-HT to *A. stephensi* in a Blood Meal

In addition to priming (see section 2.4), 5-HT was provisioned to mosquitoes via artificial blood meal for *P. falciparum* infection and for a subset of behavioral studies with uninfected *A. stephensi*. For this purpose, 5-HT was diluted in water, then added in a volume of 3 μl to 3 ml of blood meal for final concentrations of 0.15 µM and 1.5 µM 5-HT. The control blood meal was prepared identically to the others, except that 3 µl of water only was added to 3 ml of blood meal.

### 2.7 Impact of 5-HT Provisioning on Fecundity of Uninfected Female *A. stephensi* Over Time

A treated blood meal (control, 0.15 µM, and 1.5 µM 5-HT) was provided as described in section 2.6 to mosquitoes once per week. After feeding, 40 blood fed mosquitoes from each treatment group were placed individually into 50 ml conical tubes with water in the bottom and allowed 48 h to oviposit. After oviposition, females were then transferred back to group housing and the eggs were counted in each 50 ml conical tube. This process was repeated for three gonotrophic cycles with blood fed mosquitoes; non-fed mosquitoes were discarded. Separate cohorts of mosquitoes were used to perform five biological replicates.

### 2.8 Impact of 5-HT Provisioning on Lifespan and Patterns of Blood Feeding of Uninfected Female *A. stephensi* Over Time

Each of the three treatments in blood (control, 0.15 µM, and 1.5 µM 5-HT) were offered to groups of 120 female mosquitoes as described in section 2.6. To ensure all mosquitoes imbibed the first blood meal, unfed mosquitoes were removed after the first blood meal. The control and treated blood meals were offered once weekly to each group until no mosquitoes remained alive. After each weekly feeding, blood fed and non-fed mosquitoes were counted and recorded. Separate cohorts of mosquitoes were used to perform six biological replicates.

### 2.9 Impact of 5-HT Provisioning on Flight Activity and Visual Object Investigation in Response to CO_2_

All flight behavior experiments were conducted in a low-speed wind tunnel (ELD Inc., Lake City, MN, United States), as described previously (Rodriguez et al., 2021). Briefly, the wind tunnel is 224 cm long × 61 cm wide × 61 cm high and provides a constant air flow of 40 cm/s. Three projectors (LG PH450U, Englewood Cliffs, NJ, United States) provide a low contrast checkerboard on the floor of the tunnel and grey horizons on each side of the tunnel by displaying on rear projection screens (SpyeDark, Spye, LLC, Minneapolis, MN, United States). A 3D real-time tracking system (Rodriguez et al., 2021; Alonso San Alberto et al., 2022) was used to track mosquito trajectories. Sixteen cameras (Basler AC640gm, Exton, PA, United States), each with an opaque Infrared (IR) Optical Wratten Filter (Kodak 89B, Kodak, Rochester, NY, United States), were mounted on top of the wind tunnel and recorded mosquito trajectories at 60 frames/s. IR backlights (HK-F3528IR30-X, LedLightsWorld, Bellevue, WA, United States) were installed below and on the sides of the wind tunnel to provide constant illumination beyond the visual sensitivity of the mosquitoes. The ambient temperature and CO_2_ outside of the tunnel were 22.5°C and 410 ppm, respectively.

For each assay, 50 female *A. stephensi* were transferred into the tunnel and, after 1 h of acclimation, a 5% CO_2_ plume (or filtered air in control experiments) was released from the upwind section of the tunnel at a height of 30 cm in the centerline of the tunnel. The CO_2_ remained on for 1 h, after which filtered air was returned to the tunnel for 1 h (post-CO_2_). The CO_2_ and filtered air were delivered using two mass flow controllers (MC-200SCCM-D, Alicat Scientific, Tucson, AZ, United States). To examine mosquito responses to visual stimuli, we placed 5 cm diameter white and black paper circles (Color-aid Corp., Hudson Falls, NY, United States) 18 cm apart on the floor of the wind tunnel in a row perpendicular to the direction of airflow.

Our tracking system cannot maintain mosquito identities for extended periods of time, but individual trajectories were assumed to be independent for statistical analysis. Analyses were restricted to trajectories that were at least 90 frames (1.5 s) long. Trajectories that were at least 1.5 s were analyzed (average 2.07 s, longest 149.07 s, n = 3,053 trajectories). Flight velocities for each mosquito path were analyzed based on their 3D trajectory for the pre-CO_2_ (filtered air), CO_2_, and post-CO_2_ (filtered air) conditions. As described previously (Rodriguez et al., 2021), mosquito attraction to visual objects were examined using black and white circles in the tunnel. Experiments were performed with mosquitoes provisioned with 5-HT in an artificial bloodmeal (section 2.6).

### 2.10 Impact of 5-HT Provisioning on Tendency to Take a Second Blood Meal in *A. stephensi*

Mosquitoes were treated as described in section 2.4 with priming before the first blood meal or as in section 2.6 with delivery of 5-HT in the first blood meal. Following the first blood meal, non-fed mosquitoes were removed from the study. Those mosquitoes that blood fed were provided an oviposition substrate 3 days later. A second blood meal was offered at 4 days or 14 days after the first blood meal, and blood fed and non-fed mosquitoes were recorded. Each timepoint used separate cohorts of female *A. stephensi*, and five biological replicates for both 5-HT priming and 5-HT blood meal delivery were performed.

### 2.11 *Plasmodium yoelii* 17XNL Infection of *A. stephensi*

For these studies, 8–10-week-old female CD-1 mice (Envigo, Indianapolis, IN, United States) were used for *P. y. yoelii* 17XNL (hereafter referred to as *P. yoelii*) infection. Female mice were used for these studies because they are larger, easier to manipulate, and there are no distinguishable differences between male and female mice in the patterns of parasitemia and gametocytemia. Mice were maintained as described (Rodriguez et al., 2021) and all procedures were approved by the Institutional Animal Care and Use Committee of the University of Idaho (protocol IACUC-2020-10, approved 30 March 2020). Mice were infected by intraperitoneal injection of 1 × 10^7^ *P. yoelii-*infected RBCs, followed at 2 days post-infection (PI) by daily monitoring of parasitemia *via* microscopy of thin blood smears stained with Giemsa. To evaluate exflagellation of male gametocytes, wet prep slides were made from a drop of blood at 3 days PI, the day of peak transmissibility of *P. yoelii* to *A. stephensi*. Exflagellation events were counted as events per field on ×200 magnification. A total of four fields were examined per mouse and mice with 11–15 exflagellation events per field were chosen to infect mosquitoes. Each mouse was anesthetized with ketamine (50 mg/kg) and xylazine (5 mg/kg) in sterile saline and placed on top of a carton containing approximately 90 4–8 days-old female mosquitoes. Mosquitoes were allowed to feed for 15 min. After feeding was complete, mice were euthanized via CO_2_ asphyxiation followed by cervical dislocation per the approved IACUC protocol.

### 2.12 Impact of 5-HT Provisioning on Infection Success of *A. stephensi* With *P. yoelii*

Mosquitoes were primed for 3 days and infected as described in sections 2.4 and 2.11, respectively. Unfed mosquitoes were removed and discarded. Parasite infection prevalence and intensity were quantified by counting oocysts on dissected individual mercurochrome-stained midguts of 25–30 mosquitoes at 10 days post-feeding (PF). At 12-15 d PF, salivary gland pairs from each of 12–18 mosquitoes were dissected and scored on a scale of 1–4 per pair of glands, with the following system for sporozoite scores: 1 for 1–99 sporozoites, 2 for 100–999 sporozoites, 3 for 1,000–9,999 sporozoites and four for 10,000+ sporozoites. Midgut oocyst and salivary gland sporozoite infection studies were performed with six biological replicates with separate cohorts of *A. stephensi.*

### 2.13 Impact of 5-HT Provisioning on Blood Feeding Behavior of *P. yoelii*-Infected Female Mosquitoes

The six replicates prepared in section 2.12 for *P. yoelii* midgut and salivary gland infection were also used for these studies. Specifically, 15–20 blood-fed infected mosquitoes from each treatment group were housed in separate pint-sized cartons to determine treatment effect on tendency to take a subsequent blood meal at 4 days. Additional groups of 15–20 blood-fed infected mosquitoes from each treatment were housed in separate pint-sized cartons to determine treatment effect on tendency to take a subsequent blood meal at 11 days. Oviposition substrate was provided at 3 d PF and removed the following day. A second blood meal was offered at 4 days or 11 days following the infected blood meal and the numbers of fed and non-fed mosquitoes were recorded. Mosquitoes used in this assay were discarded and not used in other analyses.

### 2.14 *P. falciparum* NF54 Culture Maintenance

A parasitemia of 0.5%–0.7% was used to initiate *P. falciparum* NF54 culture. RPMI 1640 medium supplemented with 25 mM HEPES, L-glutamine, 49 mM hypoxanthine, and 8.2 mM DL-lactic acid, with 10% heat-inactivated human serum was used for parasite maintenance. Synchronization of the parasites was achieved with treatment of 5% sorbitol as described previously (Pakpour et al., 2012; Pietri et al., 2016; Souvannaseng et al., 2018; Rodriguez et al., 2021).

### 2.15 Impact of 5-HT Treatment on *P*. *falciparum* NF54 Growth *In Vitro*

*Plasmodium falciparum* NF54 culture was maintained as described above, with 0.5% ring stage parasites. Treatments were prepared in triplicate with 0.15, 1.5, and 15 µM 5-HT, and 12 nM chloroquine as a positive control for parasite killing. Plates were maintained in a candle jar with samples collected from each well immediately after treatment, at 48 h, and 96 h post treatment then stained to quantify parasitemia with flow cytometry as previously described (Rodriguez et al., 2021).

### 2.16 Impact of 5-HT on *P. falciparum* Infection Success of *A. stephensi*

*P. falciparum* NF54 culture was initiated and maintained as previously described (Rodriguez et al., 2021). On the day of feeding, exflagellation events were counted as events per field on ×200 magnification. A total of four fields were examined, and on average 4–10 events/field were observed in cultures used to infect mosquitoes. Sucrose-soaked cotton balls were removed and replaced with water-soaked cotton balls 6 h prior to the infective blood meal. Treatments of 0.15 µM and 1.5 µM 5-HT or an equivalent volume of water as a control were added to the infective blood meal, as described in section 2.6, immediately prior to feeding. After the blood meal was offered, non-fed mosquitoes were discarded. At 10 d PF, 20–30 midguts were dissected and stained with 0.5% mercurochrome. Oocysts were counted from individual midguts of mosquitoes from each of eight biological replicates. At 15 d PF, salivary glands were dissected from 7 to 15 mosquitoes from six biological replicates. Sporozoites were quantified from individual sets of salivary glands as described in section 2.12.

### 2.17 Statistical Analyses

Data were analyzed using GraphPad (version 9.3.1, San Diego, CA, United States) or MatLab (2021a release). Clutch size data were analyzed using a two-way ANOVA between treatments and within a gonotrophic cycle. The proportions of female mosquitoes laying eggs were analyzed using Chi-square test and Fisher’s exact test between groups. Individual replicates of lifespan data were analyzed using the Kaplan-Meier analysis of survival with the log-rank test to compare groups. One-way Kruskal-Wallis ANOVA with *post hoc* Tukey was used to analyze median day of survival in lifespans across replicates and median week of feeding cessation across replicates. Proportions of mosquitoes feeding over time from individual replicates were analyzed as time of feeding cessation by Kaplan-Meier analysis. Numbers of mosquitoes taking a second blood meal and infection prevalence were analyzed using Chi-square test, and Fisher’s exact test was used to compare groups. One-way Kruskal-Wallis ANOVA with *post hoc* Tukey was used to analyze infection intensity data. For flight tunnel studies, mean mosquito flight velocities were calculated from the 3D tracks of each individual trajectory and data were analyzed using Kruskal-Wallis or Mann-Whitney U-test with Bonferroni correction. For each replicate trial, the total number of flying mosquitoes and the total number of mosquitoes investigating the visual objects on the floor of the tunnel were quantified during the experimental periods (pre-CO_2_ and post-CO_2_). All data from uninfected mosquitoes and from *P. yoelii*-infected mosquitoes were analyzed by treatment using Pearson correlation matrices. Correlation matrices between treatment groups were analyzed using Procrustes analysis (PA) code from David L. Jones (http://www.rsmas.miami.edu/personal/djones/) (Rohlf and Slice, 1990; Peres-Neto et al., 2001). PA has been widely used in the field of sensory biology and relative to other multivariate analyses (i.e., PCA) does not provide dimensional reduction of large data sets through an orthogonal linear transformation. Instead, PA minimizes the sum-of-squared differences between two configurations (i.e., data matrices) in multivariate Euclidean space to match one vector to another vector through matrix translation, scaling, and rotation. Here, the vector representing the correlation for all continuous variables in response to the control treatment represented the reference matrix and the 5-HT treatments represented the rotation matrix. PA output provided a measure of the goodness of fit error (residual sum of squares error) and enabled ANOVA to determine significant differences between treatments. *P. falciparum* NF54 growth data were analyzed using ANOVA. For all analyses, significance was assumed at *p* ≤ 0.05.

## 3 Results

### 3.1 Serotonergic Innervation in the Midgut, Thoracic Ganglia, and Brain of Female *A. stephensi*

**FIGURE 1:**
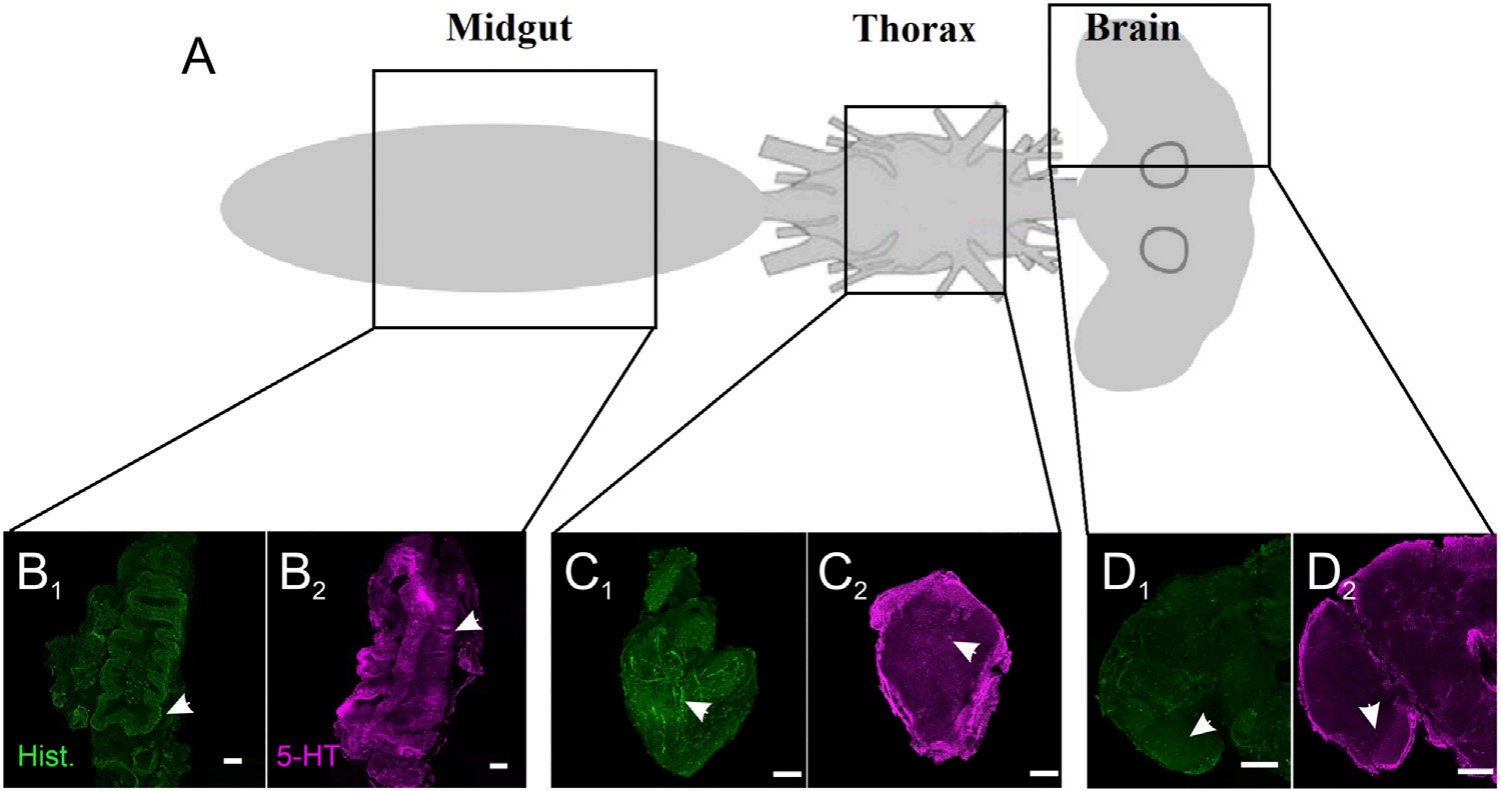
Serotonergic and histaminergic innervation of *A. stephensi* tissues. **(A)** Schematic of the regions for immunohistochemistry. **(B)** Staining of the midgut for histamine **(B**_**1**_ **)** and 5-HT **(B**_**2**_ **)**. Arrows denote the differences in staining for the lining of the midgut. **(C)** Staining of the thoracic ganglion for histamine **(C**_**1**_ **)** and 5-HT **(C**_**2**_ **)**. Arrows denote blebby-like staining. **(D)** Staining of the brain for histamine **(D**_**1**_ **)** and 5-HT **(D**_**2**_ **)**. Arrows denote stronger staining in the optic lobes for histamine and 5-HT.

As a first step to examine basal serotonergic signaling in non-fed *A. stephensi*, we stained and imaged the midgut, thoracic ganglia, and brain regions for 5-HT and histamine (Figure 1A), a biogenic amine that we examined previously (Rodriguez et al., 2021) and, therefore, a useful comparator. Histamine staining in the midgut (Figure 1B_1_) was reduced overall relative to 5-HT staining (Figure 1B_2_), with the strongest histaminergic staining in the exterior area of the midgut. By contrast, serotonergic staining in the midgut showed strong staining in the lining of the midgut (Figure 1B_2_). Differences in patterns of histaminergic and serotonergic staining were less apparent in the thoracic ganglia (Figures 1C_1_,C_2_), with fine staining of serotonergic and histaminergic neurons innervating the ganglia as well as broader staining (Figures 1C_1_,C_2_). The thoracic ganglia showed bleb-like staining of fine processes that innervated the tissue (Figures 1C_1_,C_2_). Staining of the brain showed fine processes strongly innervating the optic lobe and deutocerebrum areas of the brain (Figures 1D_1_,D_2_). Collectively, these observations suggest a tissue architecture for ingested 5-HT to signal locally and systemically in *A. stephensi*.

### 3.2 Ingested 5-HT had no Impact on *A. stephensi* Female Oviposition and Clutch Size

We examined the effects of provisioning severe malaria-associated levels of 5-HT (0.15 µM), healthy levels of 5-HT (1.5 µM) or an equivalent volume of water (control) in a weekly bloodmeal on fecundity of uninfected *A. stephensi* for 3 weeks or three gonotrophic cycles. There were no effects of treatment on clutch size nor the numbers or proportions of females that laid eggs (Supplementary Figure S1 and Supplementary Tables S1A,B).

### 3.3 Ingested 5-HT had no Impact on *A. stephensi* Lifespan and Feeding Behavior Over Time

To assess the impact of ingested 5-HT on *A. stephensi* lifespan, healthy levels of 5-HT (1.5 µM), severe malaria-associated levels of 5-HT (0.15 µM) or an equivalent volume of water were added to artificial blood meals and provisioned once weekly to uninfected female mosquitoes. Although some replicates showed significant effects of treatment (Supplementary Table S2A and Supplementary Figure S2), there were no differences in median survival time between treatments and control across all replicates (N = 6) (Supplementary Table S2B). Given that the tendency to blood feed can decline with mosquito age (Miazgowicz et al., 2020), we examined the effects of 5-HT treatment on the tendency to blood feed over the entire lifespan. Although some replicates showed significant effects of treatment (Supplementary Table S3A and Supplementary Figure S3), the median week of feeding cessation over the lifespan was not different among groups across all replicates (N = 6) (Supplementary Table S3B).

### 3.4 Ingested 5-HT Enhanced *A. stephensi* Flight Behavior in Response to Visual and Olfactory Cues

**FIGURE 2:**
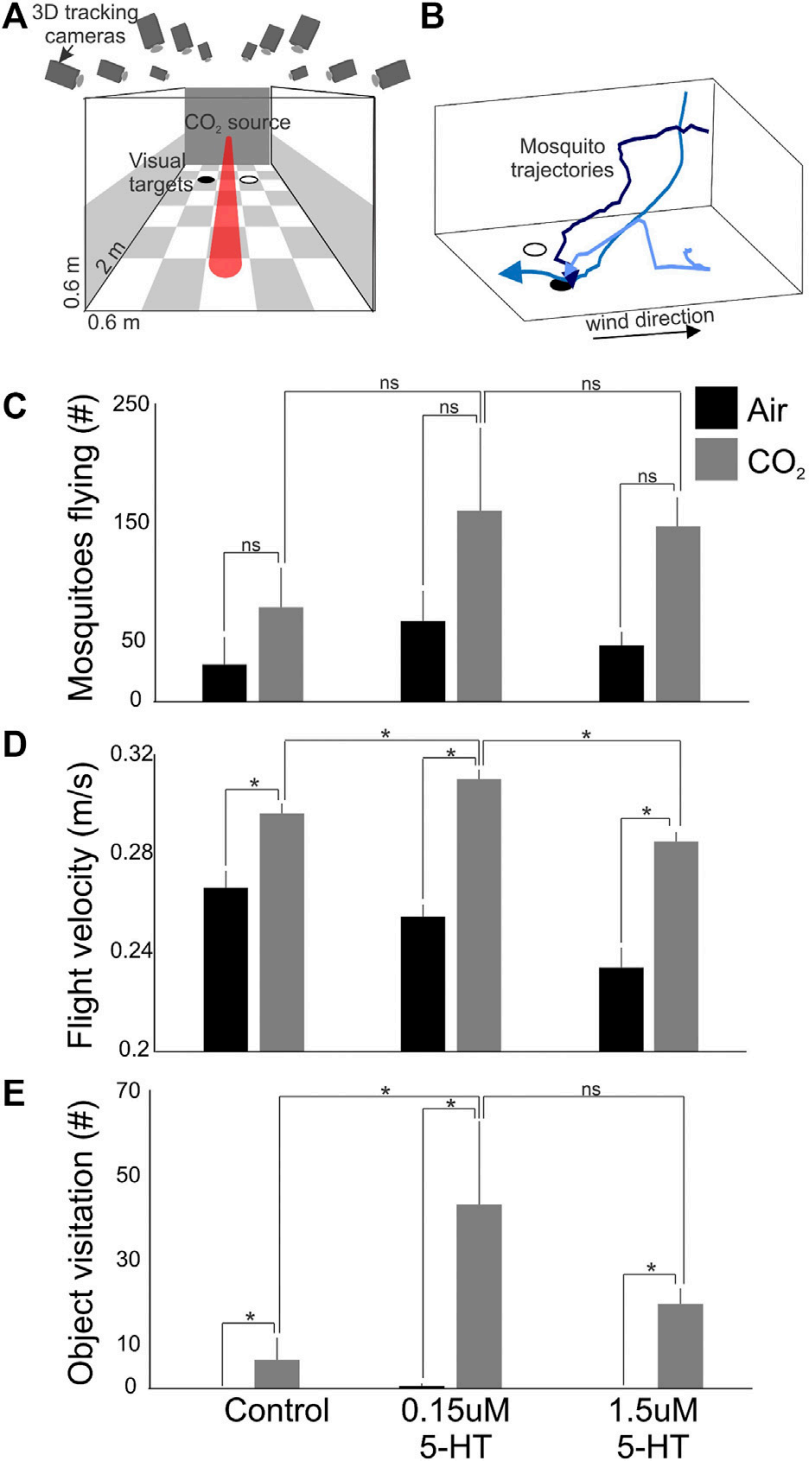
Flight activity and response to visual objects of *A. stephensi* following 5-HT provisioning in a bloodmeal. **(A)** Wind tunnel and associated computer vision system for real-time tracking of mosquito behaviors. **(B)** Example mosquito trajectories. After encountering the CO_2_ plume, mosquitoes increase their flight velocity and become sensitized to visual objects, like a black circle. **(C)** The numbers of mosquitoes flying in the wind tunnel during exposure to clean air (black histogram) and CO_2_ (grey histograms) for different treatments (control, 0.15 µM 5-HT, and 1.5 µM 5-HT). **(D)** Same as in **(C)**, except for the flight velocities of the mosquito tracks. **(E)** Same as in **(C)**, except for the numbers of mosquitoes that investigated attractive visual objects on the floor of the wind tunnel. Bars are the mean ± sem; *p < 0.05; and ns > 0.05 (Kruskal-Wallis test with Tukey *post hoc* test).

Given the patterns of 5-HT staining in the *A. stephensi* midgut and brain (Figure 1) and known effects of 5-HT on insect behavior (Novak and Rowley, 1994; Johnson et al., 2011; Ro et al., 2016; Huser et al., 2017; Ling and Raikhel, 2018; Ngai et al., 2019), we tested the effects of ingested 5-HT on *A. stephensi* flight behavior. For these studies, we provisioned *A. stephensi* with 5-HT as described in section 2.6. At 72 h post blood feeding, we flew the mosquitoes in a wind tunnel that allowed control of airflow conditions (olfactory stimuli) and visual stimuli (Figures 2A,B). 5-HT treatment altered the number of flying mosquitoes (Figure 2C), but the limited number of trials for each treatment (n = 5) likely precluded significant effects of treatment (Kruskal-Wallis test: χ^2^ = 3.47, *p* = 0.17). Nonetheless, few mosquitoes flew in the tunnel during clean air (across all replicates, mean = 49 ± 11 SEM). By contrast, CO_2_ exposure more than doubled the number of mosquitoes flying in the tunnel (across all replicates, mean = 128 ± 24 SEM). In the control group, the number of flying mosquitoes were represented by 31 and 79 mosquito tracks for the clean air and CO_2_, respectively, while tracks for mosquitoes provisioned with 0.15 µM 5-HT were approximately double that of the control at 68 and 161 mosquitoes for clean air and CO_2_, respectively (Figure 2C).

In contrast to numbers of mosquitoes flying, flight velocities were significantly different among treatments and controls (Kruskal-Wallis test: χ^2^ = 15.61, *p* = 0.0004) (Figure 2D). During CO_2_ exposure, mosquitoes provisioned with 5-HT associated with severe malaria (0.15 µM) exhibited significantly elevated flight velocities compared to controls and mosquitoes provisioned with healthy levels of 5-HT (1.5 µM) (Kruskal-Wallis with multiple comparisons, *p* < 0.05). Flight velocities of controls and mosquitoes provisioned with healthy levels of 5-HT (1.5 µM) were not significantly different (Kruskal-Wallis with multiple comparisons, *p* = 0.33) (Figure 2D). Mosquitoes provisioned with 5-HT associated with severe malaria (0.15 µM) also exhibited significantly increased visitation of visual objects relative to controls (Kruskal-Wallis test: χ^2^ = 6.01, *p* = 0.049), while there were no significant differences between controls and mosquitoes provisioned with healthy levels of 5-HT (1.5 µM) (Kruskal-Wallis with multiple comparisons, *p* = 0.26) (Figure 2E).

### 3.5 5-HT Ingested in Blood Enhanced the Tendency of Uninfected *A. stephensi* to Take a Second Blood Meal 4 days Later

**FIGURE 3:**
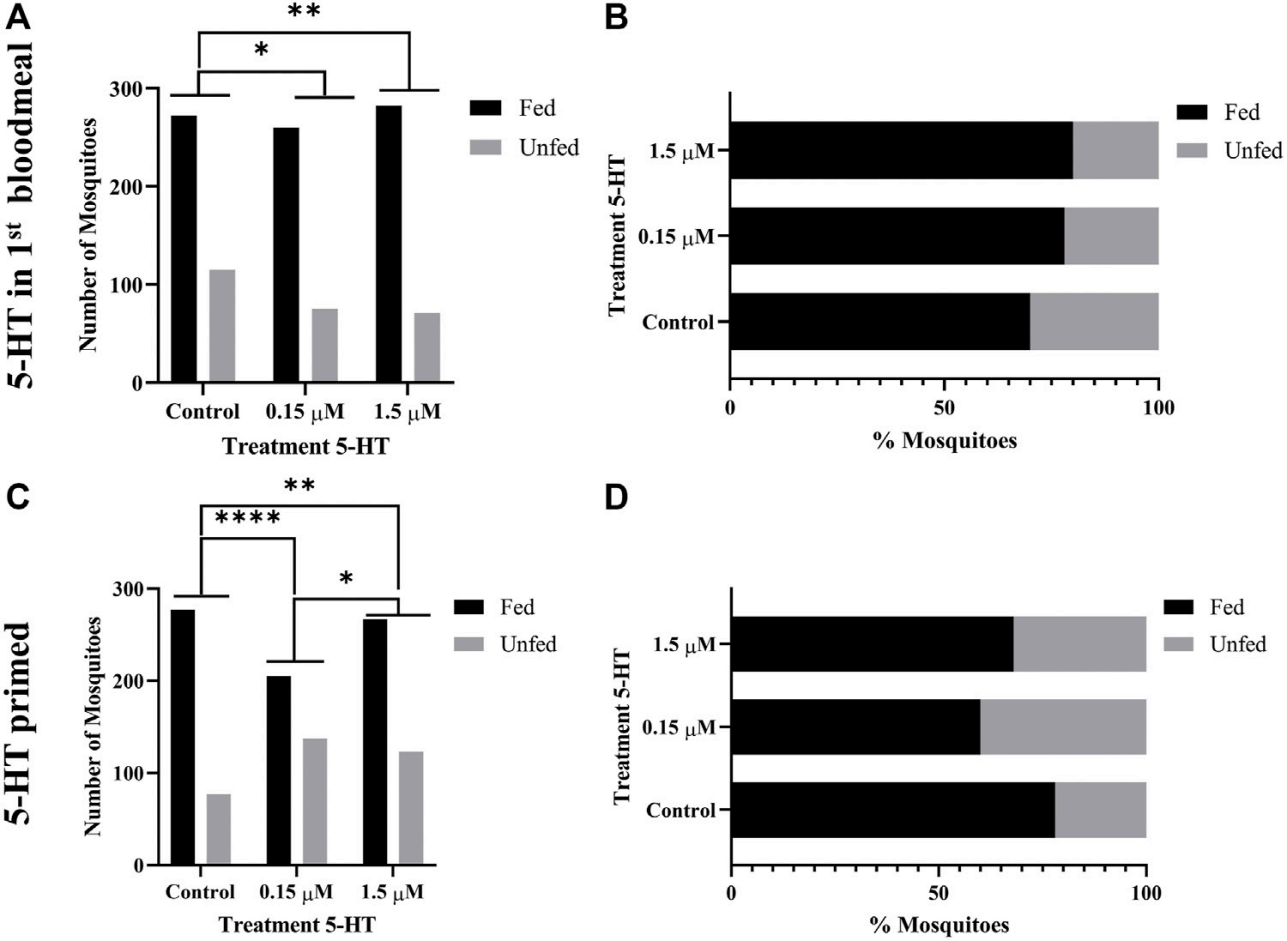
Tendency of uninfected *A. stephensi* to take a second bloodmeal 4 days later. **(A)** Numbers of fed and unfed uninfected mosquitoes provisioned with 5-HT in the first bloodmeal and **(B)** data from **(A)** shown as percentages of fed and unfed mosquitoes provisioned with 5-HT in the first bloodmeal in each group. N = 5; Fisher’s exact test (α = 0.05), *control vs. 0.15 μM *p* = 0.0277, **control vs. 1.5 μM *p* = 0.0030. **(C)** Numbers of fed and unfed uninfected mosquitoes following priming for 3 days with 5-HT in water or water only soaked cotton balls and **(D)** data from **(C)** shown as percentages of fed and unfed 5-HT primed mosquitoes in each group. N = 5; Fisher’s exact test (α = 0.05), ****control vs. 0.15 μM *p* < 0.0001, **control vs. 1.5 μM *p* = 0.0029, *0.15 μM l vs. 1.5 μM *p* = 0.0166.

**FIGURE 4:**
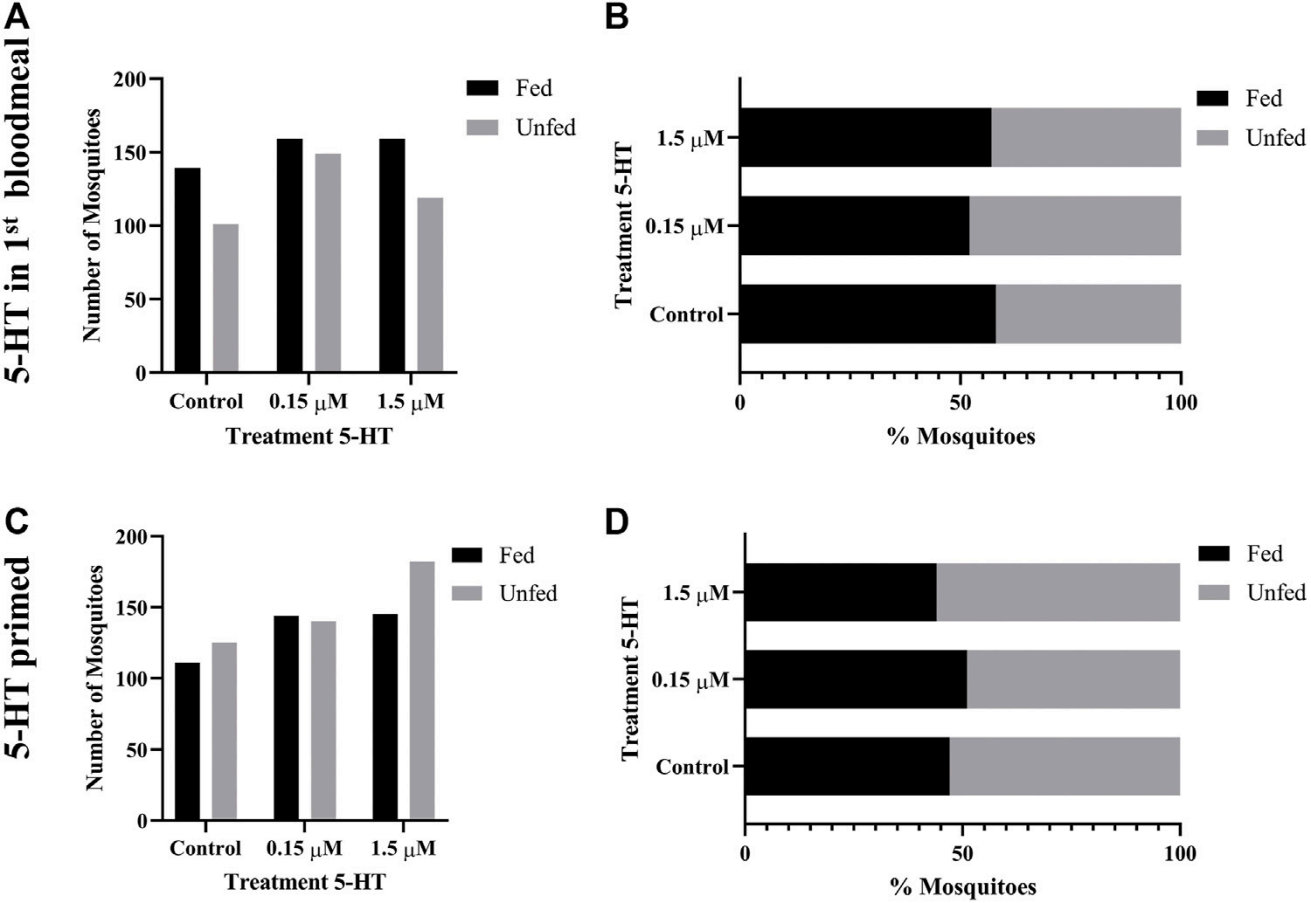
Tendency of uninfected *A. stephensi* to take a second bloodmeal 14 days later. **(A)** Numbers of fed and unfed uninfected mosquitoes provisioned with 5-HT in the first bloodmeal and **(B)** data from **(A)** shown as percentages of fed and unfed mosquitoes provisioned with 5-HT in the first bloodmeal in each group. N = 5; Chi-squared test (α = 0.05), no significance. **(C)** Numbers of fed and unfed uninfected mosquitoes following priming for 3 days with 5-HT in water or water only soaked cotton balls and **(D)** data from **(C)** shown as percentages of fed and unfed 5-HT primed mosquitoes in each group. N = 5; Chi-squared test (α = 0.05), no significance.

Given our observations of increased flight activity in response to olfactory and visual cues, we examined the effects of provisioning healthy levels of 5-HT (1.5 µM) and severe malaria-associated levels of 5-HT (0.15 µM) on the tendency of uninfected *A. stephensi* to take a second blood meal 4 days and 14 days later. Although these mosquitoes were uninfected, these timepoints were selected as markers for the midway point (4 days) and completion (14 days) of *Plasmodium* spp. sporogony. In mosquitoes provisioned with 5-HT in the first blood meal–the delivery method used for analysis of flight behavior (section 3.4) and infection with *P. falciparum* (section 3.9) – the tendency to take a second blood meal 4 days later was higher in both treatment groups compared to control (Figures 3A,B). The next set of experiments were completed by priming or provisioning 5-HT in water-soaked cotton balls for 3 days, to allow for comparison with infection studies performed with the rodent parasite model (section 3.7). The tendency to take a second blood meal 4 days later was reduced in mosquitoes primed with the severe malaria associated 5-HT (0.15 µM) compared to controls and mosquitoes primed with healthy levels of 5-HT (1.5 µM) (Figures 3C,D). Mosquitoes primed with healthy levels of 5-HT also showed a reduced tendency to blood feed 4 days later compared to controls (Figures 3C,D). There were no differences in the tendency to take a subsequent blood meal at 14 days later in mosquitoes treated with 5-HT in the first blood meal (Figures 4A,B) or in primed mosquitoes (Figures 4C,D).

### 3.6 Procrustes Analyses Revealed significant Correlations Among Biological parameters in Uninfected *A. stephensi* That Were Dependent on 5-HT Treatment

**FIGURE 5:**
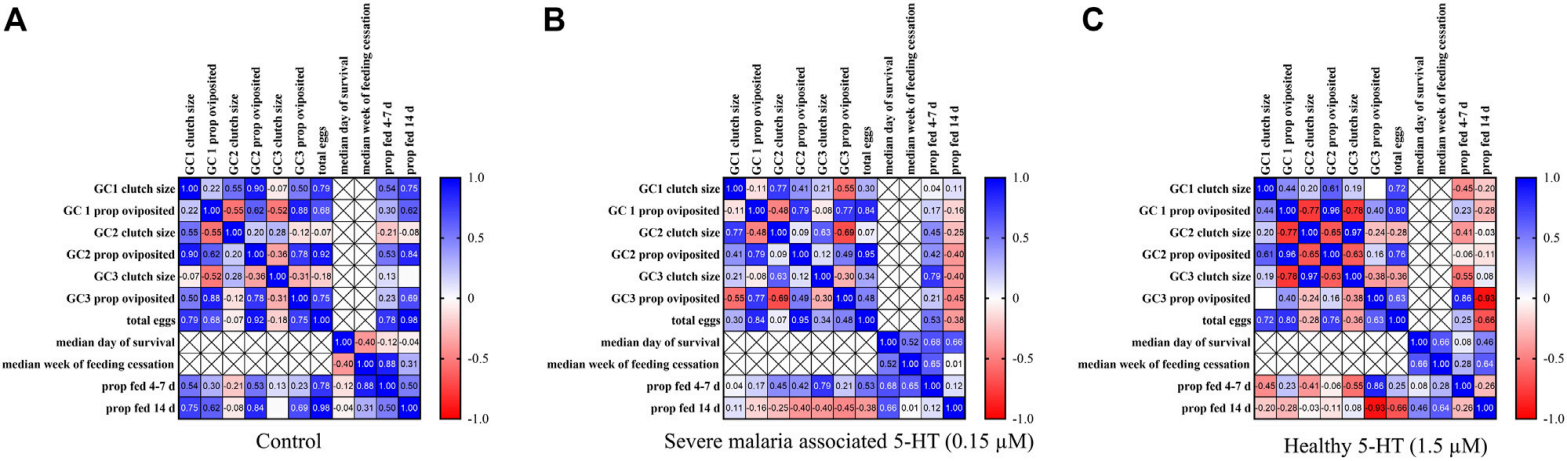
Correlation matrices of parameters from uninfected *A. stephensi* provisioned with blood meals supplemented with serotonin (5-HT) or water (control). **(A)** Correlation matrix of data from mosquitoes provisioned with blood supplemented with a volume of water (control) equivalent to that used to deliver 5-HT treatment. **(B)** Correlation matrix of data from mosquitoes provisioned with blood supplemented with severe malaria associated levels of 5-HT (0.15 µM). **(C)** Correlation matrix of data from mosquitoes provisioned with blood supplemented with healthy levels of 5-HT (1.5 µM). N = 5; Procrustes analysis, *control vs. 0.15µM, *p* = 0.0480, *control vs. 1.5µM, *p* = 0.0229, *0.15µM vs. 1.5µM, *p* = 0.0001.

We used Procrustes analysis (PA) to examine correlation patterns among parameters for uninfected mosquitoes provisioned with a control blood meal (Figure 5A) or blood meals supplemented with severe malaria associated 5-HT (0.15 µM; Figure 5B) or healthy levels of 5-HT (1.5 µM; Figure 5C). Parameters used for PA included oviposition data for gonotrophic cycles (GC) 1–3, median survival in days, median week of blood feeding cessation, and tendencies to take a second blood meal. For second blood meal, we combined 4 days data (Figures 3C,D) and 7 days data from the first week of our lifespan studies (Supplementary Figure S3). In this context, all data used for PA represented mosquitoes that fed at 4–7 days and 14 days after the first blood meal. While mosquitoes in the lifespan studies (Supplementary Tables S2A,B) were also used to assess proportion blood feeding over lifespan (Supplementary Figure S3), individual mosquitoes from lifespan studies were not assessed for oviposition. Hence, the oviposition data for GC 1-3 and lifespan data do not intersect directly and are marked as cross-hatched boxes for missing data in Figures 5A–C. Overall, PA revealed that controls were different from mosquitoes treated with 1.5 µM 5-HT (*p* = 0.0229), controls were different from mosquitoes treated with 0.15 µM 5-HT (*p* = 0.0480) and mosquitoes treated with 0.15 µM 5-HT were different from mosquitoes with 1.5 µM 5-HT (*p* = 0.0001). Controls (Figure 5A) and mosquitoes provisioned with 1.5 µM 5-HT (Figure 5C) showed similar patterns of significant positive correlations between clutch size and proportion of mosquitoes ovipositing across GC 1-3 (Supplementary Table S4). Notable differences between these two groups included a significant positive correlation between median week of feeding cessation and proportion fed at 4–7 days in controls (r = 0.88, *p* = 0.0216) and a significant negative correlation between the proportion ovipositing in GC 3 and the proportion fed at 14 days (r = −0.93, *p* = 0.0224) in mosquitoes provisioned with 1.5 µM 5-HT (Supplementary Table S4). The only significant correlation noted for mosquitoes treated with 0.15 µM 5-HT was between the proportion of mosquitoes ovipositing in GC 2 and total eggs from GC 1–3 (r = 0.95, *p* = 0.0135; Supplementary Table S4), suggesting that treatment with severe malaria 5-HT (0.15 µM) was associated with greater uncoupling of fitness parameters compared to both controls and mosquitoes provisioned with healthy levels of 5-HT (1.5 µM).

### 3.7 Treatment of *A. stephensi* With 5-HT Enhanced Infection With *P. yoelii* and Altered Blood Feeding Behavior of Infected Mosquitoes

**FIGURE 6:**
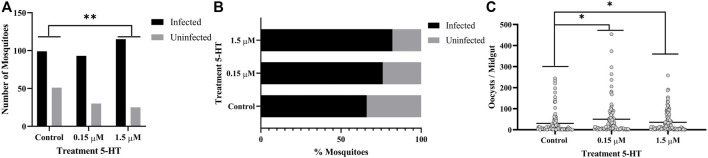
*P. yoelii yoelii* 17XNL oocyst infection in *A. stephensi* following priming for 3 days with 5-HT or water (control) soaked cotton balls. **(A)** Numbers of infected and uninfected mosquitoes by treatment with significant differences noted among pairs, and **(B)** data from **(A)** shown as percentages of uninfected and infected mosquitoes in each group. N = 6; Fisher’s exact test (α = 0.05), **control vs. 1.5 μM *p* = 0.0021. **(C)** Mean *P. y. yoelii* 17XNL midgut oocysts. N = 6; 1-way ANOVA (α = 0.05), *p* = 0.0410, *control vs. 0.15 µM, *p* = 0.0178, *control vs. 1.5 µM, *p* = 0.0490.

**FIGURE 7:**
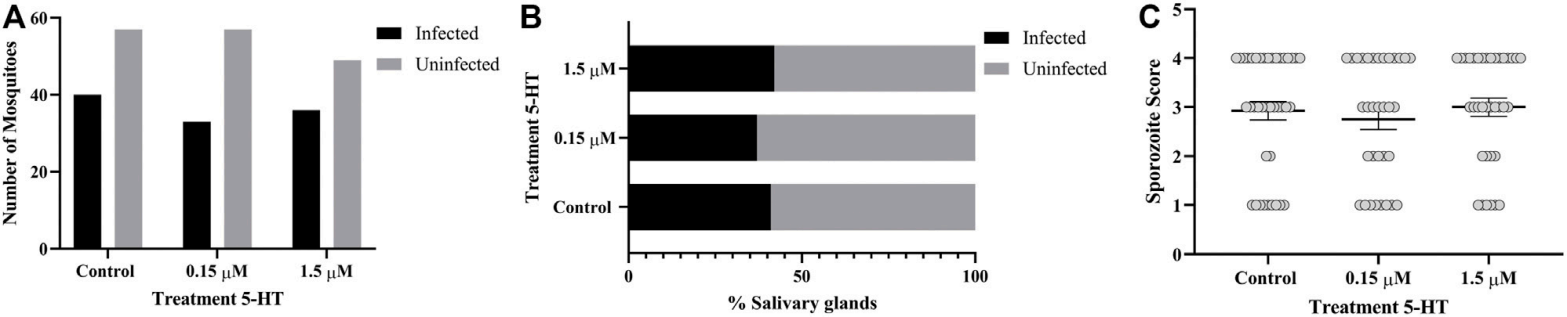
*P. yoelii yoelii* 17XNL salivary gland infection in *A. stephensi* following priming for 3 days with 5-HT or water (control) soaked cotton balls. **(A)** Numbers of mosquitoes with infected and uninfected salivary gland pairs and **(B)** data shown as percentages of uninfected and infected mosquito salivary gland pairs in each group. N = 6; Chi-squared test (α = 0.05), no significance. **(C)** Mean salivary gland score. N = 6; one-way ANOVA (α = 0.05), no significance.

Similar to humans, CD-1 mice infected with *P. yoelii* exhibit acute and sustained suppression of plasma 5-HT levels (Supplementary Figure S4). Specifically, median plasma 5-HT concentration in control, uninfected mice was 2.134 μM, while median plasma 5-HT at 2 days PI was 0.670 μM and from 4 to 10 days PI never exceeded 0.157 μM (Supplementary Figure S4). Given that peak transmissibility of *P. yoelii* to *A. stephensi* occurs at 3 days PI, mosquitoes fed on infected mice would ingest 5-HT at concentrations close to our severe malaria associated treatment of 0.15 μM 5-HT. Further, priming of *A. stephensi* with 1.5 µM 5-HT prior to feeding on infected mice would increase ingested 5-HT to levels close to that observed in the plasma of control, uninfected mice (Supplementary Figure S4). Based on these observations, we primed female *A. stephensi* for 3 days prior to feeding on *P. yoelii*-infected mice with severe malaria associated 5-HT (0.15 µM), healthy levels of 5-HT (1.5 µM) or water only in soaked cotton balls. A greater proportion of mosquitoes treated with healthy levels of 5-HT (1.5 µM) had infected midguts compared to controls (Figures 6A,B). However, mosquitoes treated with either concentration of 5-HT had significantly more oocysts per midgut than did the controls, with a trend towards higher median oocysts in mosquitoes provisioned with 0.15 µM 5-HT (median = 16) versus mosquitoes provisioned with 1.5 µM 5-HT (median = 12) (Figure 6C). There were no significant differences in the proportions of mosquitoes with sporozoite-infected salivary glands and no differences in sporozoites per salivary gland pair across controls and treatments (Figure 7).

**FIGURE 8:**
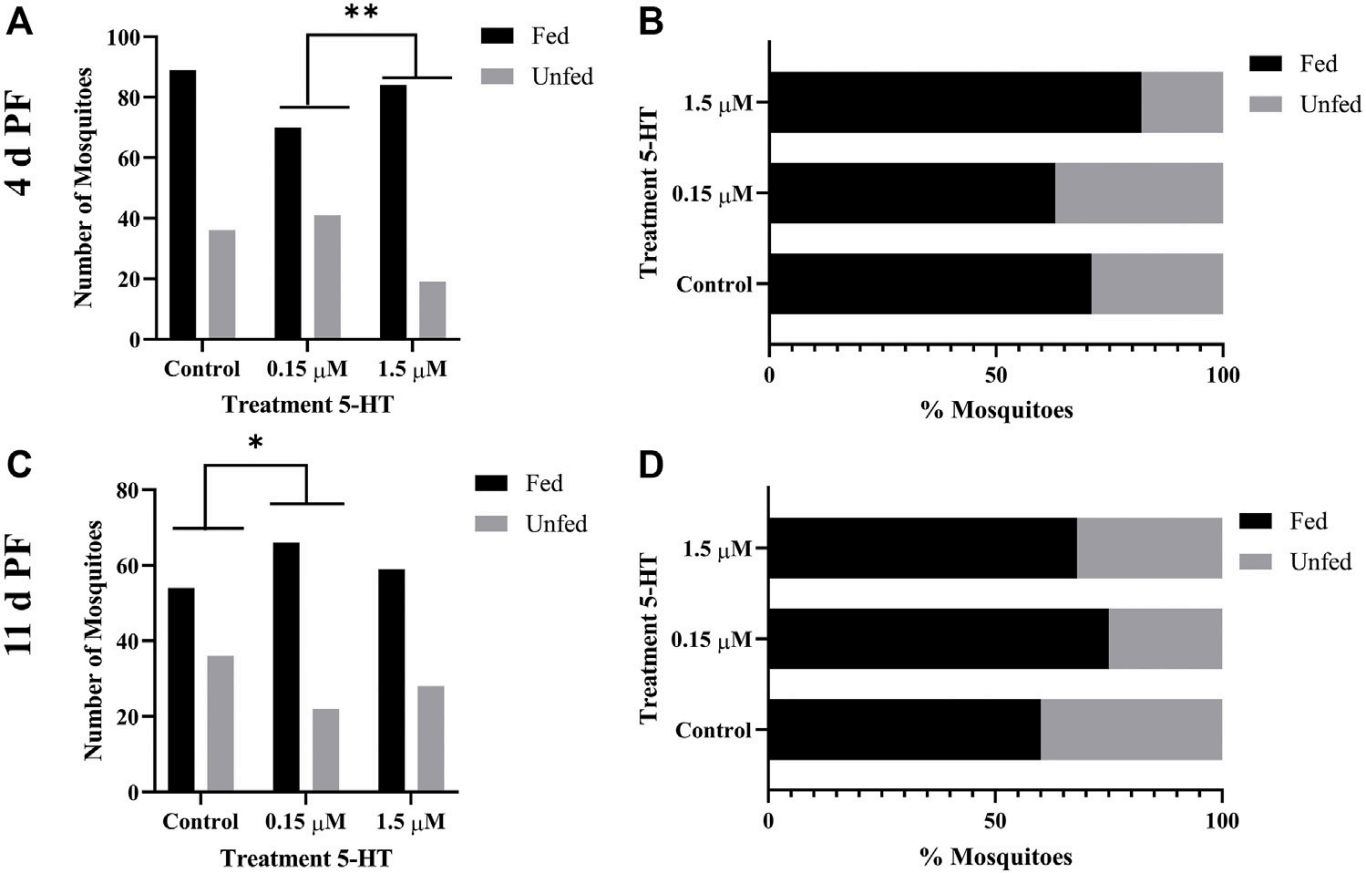
Tendency of 5-HT primed *A. stephensi* infected with *P. yoelii yoelii* 17XNL to take a second bloodmeal at 4 days or 11 days post feeding (PF). **(A)** Numbers of fed and unfed infected mosquitoes at 4 d PF and **(B)** data from **(A)** shown as percentages of fed and unfed infected mosquitoes in each group. N = 6; Fisher’s exact test (α = 0.05), **0.15 μM vs. 1.5 μM *p* = 0.0036. **(C)** Numbers of fed and unfed infected mosquitoes at 11 d PF and **(D)** data from **(C)** shown as percentages of fed and unfed infected mosquitoes in each group. N = 6; Fisher’s exact test (α = 0.05), *control vs. 0.15 μM *p* = 0.0382.

The tendency to take a second blood meal by infected mosquitoes is critical to transmission. If only oocysts are present (4 d PF), however, the risk of mosquito host death and, hence, parasite death is not offset by the potential for parasite transmission as it would be at 11 d PF, when sporozoites are present in the salivary glands. Based on this supposition, we examined the tendency of 5-HT-primed, *P. yoelii*-infected *A. stephensi* to take a second blood meal at 4 days and 11 d PF. Infected female *A. stephensi* primed with severe malaria associated 5-HT (0.15 µM) exhibited a decreased tendency to take a second blood meal at 4 d PF relative to infected mosquitoes primed with healthy levels of 5-HT (1.5 µM) (Figures 8A,B). This was similar to the decreased tendency to take a second blood meal 4 days later by uninfected *A. stephensi* following 3 days of priming and a first blood meal (Figures 3C,D). In contrast, at 11 d PF when sporozoites are present, infected *A. stephensi* provisioned with 5-HT at severe malaria concentrations (0.15 µM) exhibited an increased tendency to take a second blood meal compared to controls (Figures 8C,D). While there was a trend towards an increased tendency to take a second blood meal by infected mosquitoes primed with 0.15 µM 5-HT relative to those primed with 1.5 µM 5-HT (Figures 8C,D), this difference was not significant.

### 3.8 Procrustes Analyses Revealed significant Correlations Among Biological parameters in *P. yoelii*-Infected *A. stephensi* That Were Dependent on 5-HT Treatment

**FIGURE 9:**
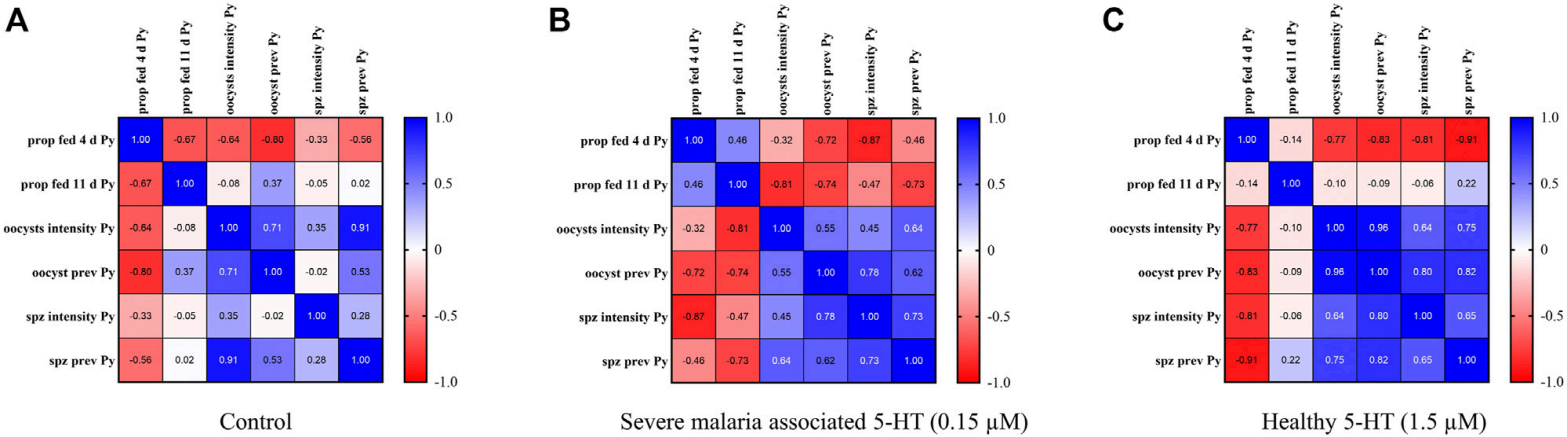
Correlation matrices of data from *A. stephensi* infected with *P. yoelii yoelii* 17XNL following priming for 3 days with 5-HT or water (control) soaked cotton balls. **(A)** Correlation matrix of data from mosquitoes treated with water (control). **(B)** Correlation matrix of data from mosquitoes treated with severe malaria associated levels of 5-HT (0.15 µM). **(C)** Correlation matrix of data from mosquitoes treated with healthy 5-HT concentrations (1.5 µM). N = 6; Procrustes analysis. *control vs. 1.5 µM, *p* = 0.0198, *0.15 µM vs. 1.5 µM, *p* = 0.0460.

We used PA to examine correlation patterns among parameters measured for *P. yoelii*-infected mosquitoes primed with water only, severe malaria associated 5-HT (0.15 µM) or healthy levels of 5-HT (1.5 µM) in soaked cotton balls. Parameters included were the tendency to take a second blood meal (proportions fed) at 4 days and 11 d PF and oocyst and sporozoite infection prevalences and intensities (Figure 9). Despite the smaller number of parameters measured for infected versus uninfected *A. stephensi* (Figure 5), PA nonetheless revealed significant differences among controls and treatments. Overall, PA revealed that controls were different from mosquitoes primed with 1.5 µM 5-HT (*p* = 0.0198) and mosquitoes primed with 0.15 µM 5-HT were different from mosquitoes primed with 1.5 µM 5-HT (*p* = 0.0460). Controls (Figure 9A) and mosquitoes primed with severe malaria associated 5-HT (0.15 µM; Figure 9C) each had a single significant correlation. For controls, oocyst infection intensity was positively correlated with sporozoite infection prevalence (r = 0.91 *p* = 0.0106), while no such correlation of oocyst and sporozoite infection patterns were observed in mosquitoes primed with severe malaria associated 5-HT (0.15 µM). The single correlation in mosquitoes primed with 0.15 µM 5-HT was represented by a negative correlation between the proportion of mosquitoes fed at 4 d PF and sporozoite infection intensity (r =−-0.87, *p* = 0.0261; Supplementary Table S4). This pattern is perhaps consistent with the finding that a second blood meal during *Plasmodium berghei* oocyst development (4 d PF) reduced oocyst numbers relative to a sugar meal in *Anopheles gambiae*, a pattern that was not observed in blood- and sugar-fed *A. gambiae* infected with *P. falciparum* (Kwon et al., 2021). In mosquitoes primed with healthy levels of 5-HT (1.5 µM) both oocyst and sporozoite prevalences were negatively correlated with the proportion fed at 4 d PF (r = −0.83, *p* = 0.0400 for oocysts; r = −0.91, *p* = 0.0115 for sporozoites), suggesting a relatively greater negative effect of a second bloodmeal at 4 d PF on parasite infection in mosquitoes ingesting healthy levels of 5-HT (1.5 µM) versus severe malaria associated 5-HT (0.15 µM).

### 3.9 Treatment of *A. stephensi* With Healthy Levels of 5-HT Reduced Infection Success With *P. falciparum*

To examine the effects of ingesting severe malaria associated 5-HT (0.15 µM) and healthy levels of 5-HT (1.5 µM) on infection of *P. falciparum* in *A. stephensi*, we added these treatments or an equivalent volume of water to a *P. falciparum* NF54 gametocyte-infected blood meal immediately prior to mosquito feeding. To ascribe the effects of 5-HT treatment in these experiments to mosquito-dependent effects on parasite development rather than to direct effects of 5-HT on parasite growth, we confirmed that *P. falciparum* growth NF54 *in vitro* was not altered by adding 5-HT directly to the parasite culture (Supplementary Figure S5). While our assays confirmed that 5-HT does not alter asexual stage *P. falciparum* growth, we cannot exclude the possibility that sexual stage parasites in the mosquito host could be directly affected by 5-HT. However, no high throughput *in vitro* assay has yet been developed for testing effects of compounds like 5-HT against all stages of sporogonic parasites, so we proceeded with these studies with the assumption that the observed effects of 5-HT would result from mosquito-dependent effects of 5-HT on *P. falciparum* development.

**FIGURE 10:**
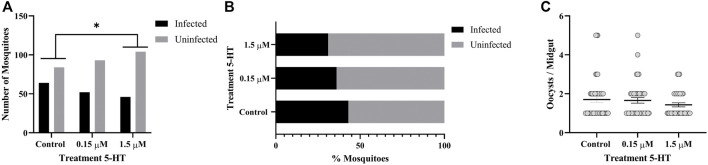
*P. falciparum* NF54 oocyst infection in *A. stephensi* following a bloodmeal provisioned with 5-HT or water (control). **(A)** Numbers of *A. stephensi* infected with *P. falciparum* NF54 oocysts, *control vs. 1.5 μM *p* = 0.0306, and **(B)** data from **(A)** shown as percentages of infected and uninfected mosquitoes in each group. N = 8; Fisher’s exact test (α = 0.05). **(C)** Mean *P. falciparum* NF54 midgut oocysts. N = 8; Kruskal-Wallace test (α = 0.05), no significance.

**FIGURE 11:**
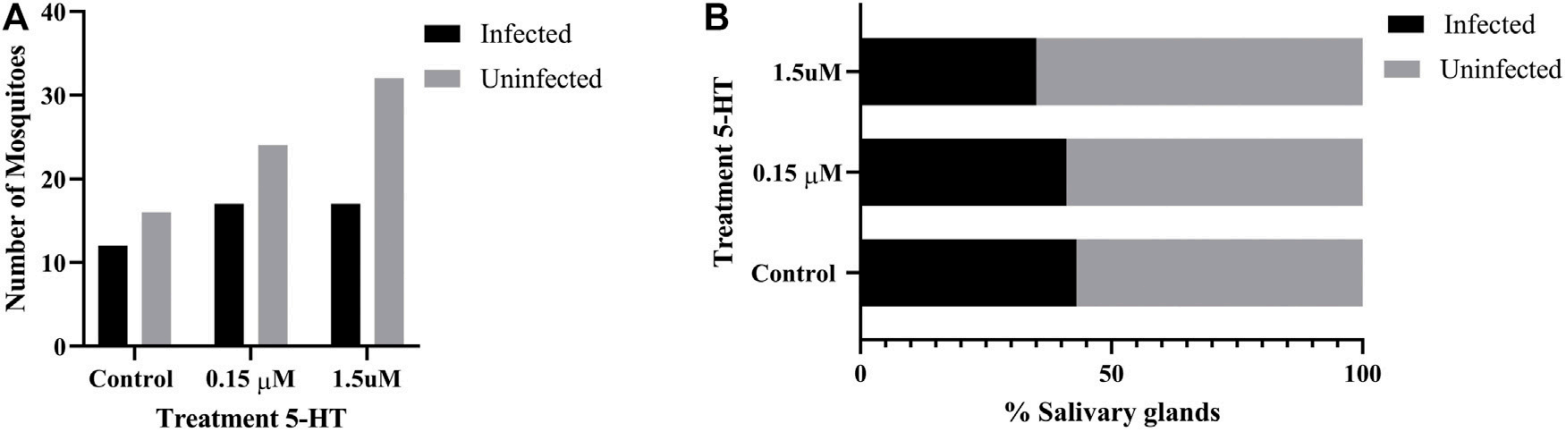
*P. falciparum* NF54 sporozoite infection in *A. stephensi* following a bloodmeal provisioned with 5-HT or water (control). **(A)** Numbers of *A. stephensi* with salivary glands infected with *P. falciparum* NF54 sporozoites and **(B)** data from **(A)** shown as percentages of sporozoite-infected and uninfected mosquitoes in each group. N = 6; Fisher’s exact test (α = 0.05), no significance.

Significantly fewer mosquitoes treated with 1.5 µM 5-HT were infected with *P. falciparum* oocysts compared to control mosquitoes (*p* = 0.0306, N = 8) (Figures 10A,B), although there were no differences in mean oocysts per midgut (Figure 10C) and no differences in proportions of mosquitoes with infected salivary glands (Figures 11A,B) across control and treatment groups. Sporozoite infection intensities for all infected control and treated mosquitoes were scored as 1 (not shown) on a scale of 1–4 (section 2.12). While there were trends towards reduced oocyst infection prevalence, mean oocysts per midgut and sporozoite infection prevalence in mosquitoes provisioned with 1.5 μM 5-HT compared to 0.15 μM 5-HT, these differences were not significant.

## 4 Discussion

Reduced blood levels of 5-HT in falciparum malaria have been associated with disease and increased gametocyte conversion (Usui et al., 2019), biology that converges on the mosquito host and parasite transmission at the interface of blood feeding. In its role as a neurotransmitter that controls a wide variety of functions in insects and other invertebrates, our observations of 5-HT in the midgut, thoracic ganglion and brain of *A. stephensi* suggest that variation in amount of ingested 5-HT could translate to patterns of mosquito physiology and behavior that impact malaria parasite transmission. Further, these effects are likely to be complex in the context of host-parasite-mosquito biology. That is, uninfected mosquitoes feeding on a gametocytemic host with severe malaria may or may not become infected (Bousema et al., 2012), but both uninfected and infected mosquitoes will have ingested blood factors including 5-HT at concentrations defined by the host disease state. Alternatively, mosquitoes already infected could subsequently feed on uninfected or infected hosts, with outcomes again defined by host health-dependent patterns of 5-HT in circulating blood. Accordingly, we tested healthy and severe malaria associated 5-HT treatments with uninfected and infected *A. stephensi*.

Neither priming nor bloodmeal delivery of severe malaria associated 5-HT altered *A. stephensi* infection prevalence with *P. yoelii* or *P. falciparum* relative to control (Figures 6, 10). The effects of healthy levels of 5-HT on *A. stephensi* infection, however, varied by both delivery method and parasite species, with increased infection prevalence relative to control for *P. yoelii* (5-HT priming) and decreased prevalence relative to control for *P. falciparum* (5-HT bloodmeal delivery) (Figures 6, 10). Notably, *P. falciparum* infection prevalence trended downward with increasing 5-HT treatment (Figure 10B), a pattern reminiscent of the inverse associations of low human blood 5-HT with high falciparum gametocyte conversion rates and high blood 5-HT with low gametocyte conversion rates (Usui et al., 2019).

With the caveat that our behavioral studies were performed only with *P. yoelii*-infected mosquitoes for safety reasons, dose effects of 5-HT on fitness and feeding behavior were notably different between uninfected and infected *A. stephensi*, suggesting that infection status of the mosquito host delineates the effects of ingested 5-HT. In uninfected *A. stephensi*, correlation analyses suggested that provisioning of severe malaria 5-HT (0.15 µM) was associated with greater uncoupling of fitness parameters compared to both controls and mosquitoes provisioned with healthy levels of 5-HT (1.5 µM; Figure 5). Further, correlation analyses suggested a relatively greater negative effect of a second bloodmeal at 4 d PF on parasite infection in mosquitoes ingesting healthy levels of 5-HT (1.5 µM) versus severe malaria associated 5-HT (0.15 µM; Figure 9). Together, these findings suggest that host blood 5-HT levels, which can be suppressed by ∼10-fold in both rodent and human malaria, impact physiology and behavior in *A. stephensi*.

Feeding behavior is of obvious importance to transmission and blood feeding behavior by *A. stephensi* was altered by ingestion of 5-HT. Our observations, however, were preceded and informed by studies in other species. In the mosquito *A. triseriatus*, oral administration (7 mM, 35 mM) or injection (4 μg) of alpha-methyl-L-tryptophan (AMTP), a modified substrate of tryptophan hydroxylase (THP) that catalyzes the first step in 5-HT biosynthesis, reduced brain 5-HT levels by >90% and reduced blood feeding success and the time to repletion (Novak and Rowley, 1994). Interestingly, the activity of THP results in the conversion of AMTP to α-methylserotonin, which like 5-HT, is an agonist for 5-HT receptors (Sourkes, 1991), so the effects of AMTP on reduced food intake in treated mice (Sivaprakasam et al., 2021), and perhaps mosquitoes, may be due to effects that are independent of 5-HT. In *A. aegypti*, blood feeding did not change levels of 5-HT in the mosquito head, with levels remaining at approximately 0.057 µM, whereas midgut 5-HT levels increased from 0.028 µM to 0.28 µM (Ling and Raikhel, 2018). In other studies, treatment of *A. aegypti* with the THP inhibitor PCPA ethyl ester at levels that were not overtly lethal to the insect were associated with increased tendency to blood feed and an increased volume of blood ingested per blood meal (Ngai et al., 2019). In our studies, the effects of 5-HT on the tendency to take a second blood meal varied by day following the first blood meal and was dependent on whether the first blood meal was uninfected or infected (Figures 3, 4, 8). Specifically, effects of priming or provisioning of 5-HT on tendency to take a second blood meal by uninfected *A. stephensi* were evident at 4 days after treatment (Figures 3, 4), while priming of *P. yoelii*-infected *A. stephensi* with 0.15 μM 5-HT was associated with effects on feeding at both 4 days and 11 d PF (Figure 8). Here, infected mosquitoes primed with severe malaria associated 5-HT (0.15 µM) exhibited a decreased tendency to take a second blood meal at 4 days and an increased tendency to take a second blood meal at 11 days (Figure 8), timepoints corresponding with oocyst and sporozoite infection, respectively, suggesting that increased transmission may be favored when malaria parasites are infective. These observations also suggest parasite infection extends the temporal effects of 5-HT on feeding of *A. stephensi*. Accordingly, the effects of 5-HT appear to be context-specific in *A. stephensi*, biology that could help to explain varied 5-HT-dependent phenotypes in other organisms.

Feeding behavior is also dependent on gut physiology and the effects of 5-HT on gut physiology in more ancient organisms and non-blood feeding insects suggest that adaptation to blood-derived 5-HT was enabled by this biology. In *Caenorhabditis elegans*, *tph-1* deletion and inhibition of 5-HT biosynthesis were shown to reduce contractions in the gut, a phenotype associated with reduced feeding rate (Szø et al., 2000). In *D. melanogaster*, addition of 5-HT to the abdomen increased contractions of the crop, while addition of 5-HT to the head decreased contractions of the crop compared to control (Solari et al., 2017). In the ant *Camponotus mus*, ingestion of increasing levels of 5-HT in sugar water (0.75 mM, 7.5 mM, and 75 mM) dose-dependently reduced food intake (Falibene et al., 2012). *D. melanogaster* and *C. mus* ingest plant material–rotting fruit and nectar, respectively–both of which contain 5-HT. Indeed, fruits, seeds and vegetables are significant sources of 5-HT (Briguglio et al., 2018; Mukherjee, 2018) as are many protists, yeasts and bacteria (Danilovich et al., 2021), suggesting that general omnivory and perhaps microbial colonization may have provided the fundamental basis for adaptation to host blood 5-HT in hematophagous arthropods. In this context, an understanding of the effects of ingested 5-HT on endogenous 5-HT in *A. stephensi* will be necessary to interpret the phenotypes that we have observed.

We observed significant effects of treatment with severe malaria associated levels of 5-HT on flight velocity and investigation of visual objects in response to host odors by *A. stephensi* (Figure 2). These behaviors were consistent with tendency to take a second blood meal at 4 days in uninfected mosquitoes (Figure 3), are new to our understanding of *A. stephensi* as a vector and are informed by studies of serotonergic signaling of locomotion and sensory processing in other insects. In *D. melanogaster*, walking speed was decreased when ventral nerve cord serotonergic signaling was activated, but walking speed was increased when this signaling was inhibited (Howard et al., 2019). In the honeybee *Apis mellifera*, however, flight behavior was not impacted by manipulation of 5-HT (Zhao et al., 2014). In *A. aegypti*, reduced 5-HT levels were associated with increased flight distance (Ngai et al., 2019). The effects of 5-HT on mosquito olfaction and vision could be reflected in the processing of this sensory information. In *D. melanogaster*, 5-HT plays an important role in gain control in the fly antennal lobe, and serotonergic neurons are predominantly located and innervate the fly optic lobe suggesting important functional effects (Bao et al., 2010; Suzuki et al., 2020). It remains unclear whether the ingestion of the 5-HT in the blood meal could mediate similar functional effects in the *A. stephensi* brain, but passage of ingested 5-HT into the body with circulation to the brain or a neuromodulatory link suggested by our immunohistochemistry data could mediate gut-to-brain and brain-to-gut 5-HT signaling in *A. stephensi*.

We observed no significant effects of 5-HT on reproduction or lifespan of *A. stephensi*. Studies in other organisms provide insights and comparisons. Treatment of *D. melanogaster* with 38 mM and 75 mM 5-HT, concentrations more than 10^4^ times higher than those used in our studies, decreased fly oviposition success (Thomas et al., 1998). Treatment of *C. elegans* with a similarly high concentrations of 5-HT (7.5 mM and 57 mM) depressed locomotion and altered egg laying rate, but did not impact the number of eggs laid (Horvitz et al., 1982; Waggoner et al., 1998). In contrast, targeted deletion of *tph-1* in *C. elegans* inhibited 5-HT biosynthesis and increased reproductive lifespan (Szø et al., 2000). In agreement with our studies, manipulations to increase and decrease 5-HT levels in *C. elegans* had no effect on nematode lifespan (Murakami and Murakami, 2007). Collectively, these studies suggest that effects on reproduction and lifespan are most accurately assessed at concentrations of 5-HT that are physiological to the host.

## 5 Conclusion and Perspectives

5-HT is one of numerous compounds in human blood that varies in concentration with malaria and that can signal to the mosquito host following blood feeding (Pakpour et al., 2013; Pakpour et al., 2014; Luckhart et al., 2015; Luckhart and Riehle, 2017). Hence, 5-HT does not alter mosquito physiology in isolation. In the studies herein, we observed distinct patterns and intensity of 5-HT and histamine staining in the *A. stephensi* midgut, but similar patterns of staining in the thoracic ganglion and brain, proximity and patterns that suggest coordination of 5-HT and histamine signaling beyond the midgut (Figure 1). Intriguingly, 5-HT activates histaminergic signaling in rat brain neurons (Sergeeva et al., 2003) and, in mice, integration of 5-HT and histamine signaling regulates food intake (Murotani et al., 2011). In previous studies, we showed that priming and provisioning of *A. stephensi* with severe malaria-associated histamine levels (10 nM) relative to control mosquitoes and mosquitoes treated with healthy levels of histamine (1 nM) significantly increased mosquito flight activity, blood feeding over the course of mosquito lifespan, infection with both *P. yoelii* and *P. falciparum*, and the tendency of uninfected (but not infected) mosquitoes to take a second blood meal. While these effects were similar to those observed with 5-HT, they were not identical, suggesting that some level of 5-HT and histamine signaling integration contributes to the observed patterns in *A. stephensi*. Accordingly, we are currently examining this possibility and expanding our understanding of the integration of metabolism, behavior, and host defenses to include these novel aspects of neurophysiology in *A. stephensi*.

## Data Availability

The datasets presented in this study can be found in online repositories. The names of the repository/repositories and accession number(s) can be found below: https://github.com/riffelllab (https://doi.org/10.5281/zenodo.5579784).

## References

[B1] Alonso San Alberto D. Rusch C. Zhan Y. Straw A. D. Craig M. Riffell J. A. (2022). The Olfactory Gating of Visual Preferences to Human Skin and Colors in Mosquitoes. Nat. Commun. 13 (1), 555. 10.1038/s41467-022-28195-x 35121739 PMC8816903

[B2] Badcock N. R. Spence J. G. Stern L. M. (1987). Blood Serotonin Levels in Adults, Autistic and Non-autistic Children-With a Comparison of Different Methodologies. Ann. Clin. Biochem. 24 (6), 625–634. 10.1177/000456328702400613 3426129

[B3] Bao X. Wang B. Zhang J. Yan T. Yang W. Jiao F. (2010). Localization of Serotonin/Tryptophan-Hydroxylase-Immunoreactive Cells in the Brain and Suboesophageal Ganglion of *Drosophila melanogaster* . Cell Tissue Res. 340 (1), 51–59. 10.1007/s00441-010-0932-5 20177707

[B4] Bousema T. Dinglasan R. R. Morlais I. Gouagna L. C. van Warmerdam T. Awono-Ambene P. H. (2012). Mosquito Feeding Assays to Determine the Infectiousness of Naturally Infected *Plasmodium Falciparum* Gametocyte Carriers. PLoS ONE 7, e42821. 10.1371/journal.pone.0042821 22936993 PMC3425579

[B5] Bradley S. P. Chapman P. D. Lizbinski K. M. Daly K. C. Dacks A. M. (2016). A Flight Sensory-Motor to Olfactory Processing Circuit in the Moth *Manduca Sexta* . Front. Neural Circuits 10, 5. 10.3389/fncir.2016.00005 26909026 PMC4754697

[B6] Briguglio M. Dell’Osso B. Panzica G. Malgaroli A. Banfi G. Zanaboni Dina C. (2018). Dietary Neurotransmitters: A Narrative Review on Current Knowledge. Nutrients 10, 591. 10.3390/nu10050591 PMC5986471 29748506

[B7] Chapman P. D. Bradley S. P. Haught E. J. Riggs K. E. Haffar M. M. Daly K. C. (2017). Co-Option of a Motor-To-Sensory Histaminergic Circuit Correlates with Insect Flight Biomechanics. Proc. R. Soc. B 284, 20170339. 10.1098/rspb.2017.0339 PMC5543211 28747471

[B8] Chapman P. D. Burkland R. Bradley S. P. Houot B. Bullman V. Dacks A. M. (2018). Flight Motor Networks Modulate Primary Olfactory Processing in the Moth *Manduca Sexta* . Proc. Natl. Acad. Sci. U.S.A. 115, 5588–5593. 10.1073/pnas.1722379115 29735707 PMC6003457

[B9] Danilovich M. E. Alberto M. R. Juárez Tomás M. S. (2021). Microbial Production of Beneficial Indoleamines (Serotonin and Melatonin) with Potential Application to Biotechnological Products for Human Health. J. Appl. Microbiol. 131, 1668–1682. 10.1111/jam.15012 33484616

[B10] Dobbie M. Marsh K. Crawley J. Surtees R. Waruiru C. (2000). Cerebrospinal Fluid Studies in Children with Cerebral Malaria: An Excitotoxic Mechanism? Am. J. Trop. Med. Hyg. 62 (2), 284–290. 10.4269/ajtmh.2000.62.284 10813486

[B11] Enwonwu C. O. Afolabi B. M. Salako L. A. Idigbe E. O. Al-Hassan H. Rabiu R. A. (1999). Hyperphenylalaninaemia in Children with Falciparum Malaria. Qjm 92, 495–503. 10.1093/qjmed/92.9.495 10627868

[B12] Falibene A. Rössler W. Josens R. (2012). Serotonin Depresses Feeding Behaviour in Ants. J. Insect Physiology 58 (1), 7–17. 10.1016/j.jinsphys.2011.08.015 21893064

[B13] World Health Organization (2020). World Malaria Report 2020: 20 Years of Global Progress and Challenges. Geneva, Switzerland: World Health Organization. Available at: https://apps.who.int/iris/handle/10665/337660 .

[B14] Horvitz H. R. Chalfie M. Trent C. Sulston J. E. Evans P. D. (1982). Serotonin and Octopamine in the Nematode *Caenorhabditis elegans* . Science 216 (4549), 1012–1014. 10.1126/science.6805073 6805073

[B15] Howard C. E. Chen C.-L. Tabachnik T. Hormigo R. Ramdya P. Mann R. S. (2019). Serotonergic Modulation of Walking in *Drosophila* . Curr. Biol. 29 (24), 4218–4230. 10.1016/j.cub.2019.10.042 31786064 PMC6935052

[B16] Huser A. Eschment M. Güllü N. Collins K. A. N. Böpple K. Pankevych L. (2017). Anatomy and Behavioral Function of Serotonin Receptors in *Drosophila melanogaster* Larvae. PLoS ONE 12 (8), e0181865. 10.1371/journal.pone.0181865 28777821 PMC5544185

[B17] Johnson O. Becnel J. Nichols C. D. (2011). Serotonin Receptor Activity Is Necessary for Olfactory Learning and Memory in *Drosophila melanogaster* . Neuroscience 192, 372–381. 10.1016/j.neuroscience.2011.06.058 21749913 PMC3166404

[B18] Kwon H. Simões M. L. Reynolds R. A. Dimopoulos G. Smith R. C. (2021). Additional Feeding Reveals Differences in Immune Recognition and Growth of Plasmodium Parasites in the Mosquito Host. mSphere 6 (2), e00136. 10.1128/mSphere.00136-21 33789941 PMC8546690

[B19] Ling L. Raikhel A. S. (2018). Serotonin Signaling Regulates Insulin-like Peptides for Growth, Reproduction, and Metabolism in the Disease *Vector Aedes aegypti* . Proc. Natl. Acad. Sci. U.S.A. 115 (42), E9822. 10.1073/pnas.1808243115 30275337 PMC6196551

[B20] Loo Y. H. (1974). Serotonin Deficiency in Experimental Hyperphenylalaninemia. J. Neurochem. 23, 139–147. 10.1111/j.1471-4159.1974.tb06928.x 4546911

[B21] Lopansri B. K. Anstey N. M. Stoddard G. J. Mwaikambo E. D. Boutlis C. S. Tjitra E. (2006). Elevated Plasma Phenylalanine in Severe Malaria and Implications for Pathophysiology of Neurological Complications. Infect. Immun. 74, 3355–3359. 10.1128/IAI.02106-05 16714564 PMC1479261

[B22] Luckhart S. Pakpour N. Giulivi C. (2015). Host-Pathogen Interactions in Malaria: Cross-Kingdom Signaling and Mitochondrial Regulation. Curr. Opin. Immunol. 36, 73–79. 10.1016/j.coi.2015.07.002 26210301 PMC4593738

[B23] Luckhart S. Riehle M. A. (2017). Conservation and Convergence of Immune Signaling Pathways with Mitochondrial Regulation in Vector Arthropod Physiology. Arthropod Vector Control. Dis. Transm. 1, 15–33. 10.1016/B978-0-12-805350-8.00002-7

[B24] Miazgowicz K. L. Shocket M. S. Ryan S. J. Villena O. C. Hall R. J. Owen J. (2020). Age Influences the Thermal Suitability of *Plasmodium Falciparum* Transmission in the Asian Malaria Vector *Anopheles stephensi* . Proc. R. Soc. B 287, 20201093. 10.1098/rspb.2020.1093 PMC7423674 32693720

[B25] Mukherjee S. (2018). Novel Perspectives on the Molecular Crosstalk Mechanisms of Serotonin and Melatonin in Plants. Plant Physiology Biochem. 132, 33–45. 10.1016/j.plaphy.2018.08.031 30172851

[B26] Murakami H. Murakami S. (2007). Serotonin Receptors Antagonistically modulateCaenorhabditis Eleganslongevity. Aging Cell 6 (4), 483–488. 10.1111/j.1474-9726.2007.00303.x 17559503

[B27] Murotani T. Ishizuka T. Isogawa Y. Karashima M. Yamatodani A. (2011). Possible Involvement of Serotonin 5-HT2 Receptor in the Regulation of Feeding Behavior through the Histaminergic System. Neuropharmacology 61 (1–2), 228–233. 10.1016/j.neuropharm.2011.04.003 21514311

[B28] Ngai M. Shoue D. A. Loh Z. McDowell M. A. (2019). The Pharmacological and Functional Characterization of the Serotonergic System in *Anopheles gambiae* and *Aedes aegypti*: Influences on Flight and Blood-Feeding Behavior. Sci. Rep. 9 (1), 1–10. 10.1038/s41598-019-38806-1 30872615 PMC6418270

[B29] Nishanth G. Schlüter D. (2019). Blood-Brain Barrier in Cerebral Malaria: Pathogenesis and Therapeutic Intervention. Trends Parasitol. 35, 516–528. 10.1016/j.pt.2019.04.010 31147271

[B30] Novak M. G. Rowley W. A. (1994). Serotonin Depletion Affects Blood-Feeding but Not Host-Seeking Ability in *Aedes triseriatus* (Diptera: Culieidae). J. Med. Entomology 31 (4), 600–606. 10.1093/jmedent/31.4.600 7932607

[B31] O’Donnell A. J. Rund S. S. C. Reece S. E. (2019). Time-of-Day of Blood-Feeding: Effects on Mosquito Life History and Malaria Transmission. Parasites Vectors 12, 301. 10.1186/s13071-019-3513-9 31262362 PMC6604169

[B32] Pakpour N. Akman-Anderson L. Vodovotz Y. Luckhart S. (2013). The Effects of Ingested Mammalian Blood Factors on Vector Arthropod Immunity and Physiology. Microbes Infect. 15, 243–254. 10.1016/j.micinf.2013.01.003 23370408 PMC3602389

[B33] Pakpour N. Corby-Harris V. Green G. P. Smithers H. M. Cheung K. W. Riehle M. A. (2012). Ingested Human Insulin Inhibits the Mosquito NF-κb-dependent Immune Response to Plasmodium Falciparum. Infect. Immun. 80, 2141–2149. 10.1128/IAI.00024-12 22473605 PMC3370580

[B34] Pakpour N. Riehle M. A. Luckhart S. (2014). Effects of Ingested Vertebrate-Derived Factors on Insect Immune Responses. Curr. Opin. Insect Sci. 3, 1–5. 10.1016/j.cois.2014.07.001 25401083 PMC4228800

[B35] Peres-Neto P. R. Jackson D. A. (2001). How Well Do Multivariate Data Sets Match? the Advantages of a Procrustean Superimposition Approach over the Mantel Test. Oecologia 129 (2), 169–178. 10.1007/s004420100720 28547594

[B36] Pietri J. E. Pakpour N. Napoli E. Song G. Pietri E. Potts R. (2016). Two Insulin-like Peptides Differentially Regulate Malaria Parasite Infection in the Mosquito through Effects on Intermediary Metabolism. Biochem. J. 473, 3487–3503. 10.1042/BCJ20160271 27496548 PMC5508860

[B37] Ro J. Pak G. Malec P. A. Lyu Y. Allison D. B. Kennedy R. T. (2016). Serotonin Signaling Mediates Protein Valuation and Aging. ELife 5, e16843. 10.7554/eLife.16843 27572262 PMC5005037

[B38] Rodriguez A. M. Hambly M. G. Jandu S. Simão-Gurge R. Lowder C. Lewis E. E. (2021). Histamine Ingestion by *Anopheles stephensi* Alters Important Vector Transmission Behaviors and Infection Success with Diverse *Plasmodium* Species. Biomolecules 11 (5), 719. 10.3390/biom11050719 34064869 PMC8151525

[B39] Rohlf F. J. Slice D. (1990). Extensions of the Procrustes Method for the Optimal Superimposition of Landmarks. Syst. Zool. 39 (1), 40. 10.2307/2992207

[B40] Sergeeva O. A. Amberger B. T. Eriksson K. S. Scherer A. Haas H. L. (2003). Co-Ordinated Expression of 5-HT2C Receptors with the NCX1 Na+/Ca2+ Exchanger in Histaminergic Neurones. J. Neurochem. 87 (3), 657–664. 10.1046/j.1471-4159.2003.02036.x 14535948

[B41] Sinka M. E. Pironon S. Massey N. C. Longbottom J. Hemingway J. Moyes C. L. (2020). A New Malaria Vector in Africa: Predicting the Expansion Range of *Anopheles stephensi* and Identifying the Urban Populations at Risk. Proc. Natl. Acad. Sci. U.S.A. 117, 24900–24908. 10.1073/pnas.2003976117 32929020 PMC7547157

[B42] Sivaprakasam S. Ramachandran S. Sikder M. O. F. Bhutia Y. D. Wachtel M. W. Ganapathy V. (2021). α-Methyl-l-tryptophan as a Weight-Loss Agent in Multiple Models of Obesity in Mice. Biochem. J. 478 (7), 1347–1358. 10.1042/BCJ20210100 33720280 PMC8038855

[B43] Solari P. Rivelli N. De Rose F. Picciau L. Murru L. Stoffolano J. G. (2017). Opposite Effects of 5-HT/AKH and Octopamine on the Crop Contractions in Adult *Drosophila melanogaster*: Evidence of a Double Brain-Gut Serotonergic Circuitry. PLoS ONE 12 (3), e0174172. 10.1371/journal.pone.0174172 28334024 PMC5363830

[B44] Sourkes T. L. (1991). Alpha-Methyltryptophan as a Therapeutic Agent. Prog. Neuropsychopharmacol. Biol. Psychiatry 15 (6), 935. 10.1016/0278-5846(91)90020-2 1763198

[B45] Souvannaseng L. Hun L. V. Baker H. Klyver J. M. Wang B. Pakpour N. (2018). Inhibition of JNK Signaling in the Asian Malaria Vector *Anopheles stephensi* Extends Mosquito Longevity and Improves Resistance to *Plasmodium Falciparum* Infection. PLoS Pathog. 14, e1007418. 10.1371/journal.ppat.1007418 30496310 PMC6264519

[B46] Suzuki Y. Schenk J. E. Tan H. Gaudry Q. (2020). A Population of Interneurons Signals Changes in the Basal Concentration of Serotonin and Mediates Gain Control in the *Drosophila* Antennal Lobe. Curr. Biol. 30 (6), 1110–1118. 10.1016/j.cub.2020.01.018 32142699 PMC7133499

[B47] Sze J. Y. Victor M. Loer C. Shi Y. Ruvkun G. (2000). Food and Metabolic Signalling Defects in a *Caenorhabditis elegans* Serotonin-Synthesis Mutant. Nature 403 (6769), 560–564. 10.1038/35000609 10676966

[B48] Thomas J. C. Saleh E. F. Alammar N. Akroush A. M. (1998). The Indole Alkaloid Tryptamine Impairs Reproduction in *Drosophila melanogaster* . J. Econ. Entomology 91 (4), 841–846. 10.1093/jee/91.4.841 9725032

[B49] Usui M. Prajapati S. K. Ayanful-Torgby R. Acquah F. K. Cudjoe E. Kakaney C. (2019). Publisher Correction: *Plasmodium Falciparum* Sexual Differentiation in Malaria Patients Is Associated with Host Factors and GDV1-dependent Genes. Nat. Commun. 10 (1), 2740. 10.1038/s41467-019-10805-w 31227704 PMC6588616

[B50] Waggoner L. E. Zhou G. T. Schafer R. W. Schafer W. R. (1998). Control of Alternative Behavioral States by Serotonin in *Caenorhabditis elegans* . Neuron 21 (1), 203–214. 10.1016/S0896-6273(00)80527-9 9697864

[B51] Winn S. R. Scherer T. Thöny B. Ying M. Martinez A. Weber S. (2018). Tanja Scherer, Beat Thöny, Ming Ying, Aurora Martinez, Sydney Weber, Jacob Raber, and Cary O. HardingBlood Phenylalanine Reduction Corrects CNS Dopamine and Serotonin Deficiencies and Partially Improves Behavioral Performance in Adult Phenylketonuric Mice. Mol. Genet. Metabolism 123 (1), 6–20. 10.1016/j.ymgme.2017.10.009 PMC5786171 29331172

[B52] Zhao H. Zheng N. Ribi W. A. Zheng H. Xue L. Gong F. (2014). Neuromechanism Study of Insect-Machine Interface: Flight Control by Neural Electrical Stimulation. PLoS ONE 9 (11), e113012. 10.1371/journal.pone.0113012 25409523 PMC4237392

