# Supplementary material for: Anopheles stephensi Feeding, Flight Behavior, and Infection With Malaria Parasites are Altered by Ingestion of Serotonin
Source: Front Physiol. 2022 Jun 7;13:911097. doi: 10.3389/fphys.2022.911097 (PMC9209645; doi:10.3389/fphys.2022.911097)
Supplement: Supplementary file 1 [file DataSheet1.docx]

Supplementary Material

# Supplementary Data

## Supplementary Figures


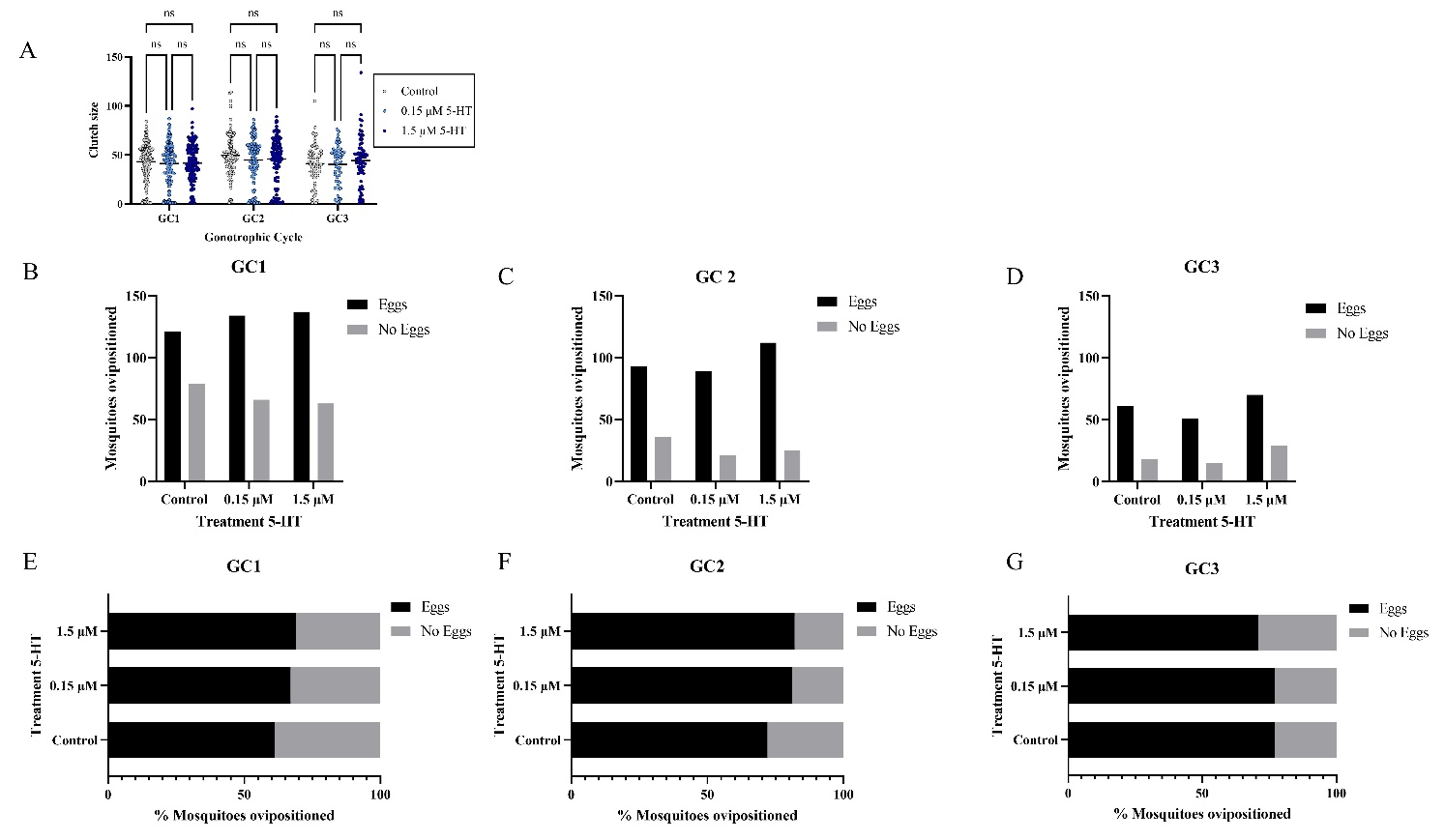


Supplementary Figure 1. Impact of ingested 5-HT in a weekly bloodmeal on *A. stephensi* reproduction. (A) Clutch size of individual *A. stephensi* females during three gonotrophic cycles (GC). N = 5, two-way ANOVA, no significance. The number of mosquitoes that oviposited or did not oviposit after (B) GC1, (C) GC2, and (D) GC3. The proportion of mosquitoes that oviposited or did not oviposit after (E) GC1, (F) GC2, and (G) GC3. N = 5; Chi-squared test (α = 0.05), no significance.


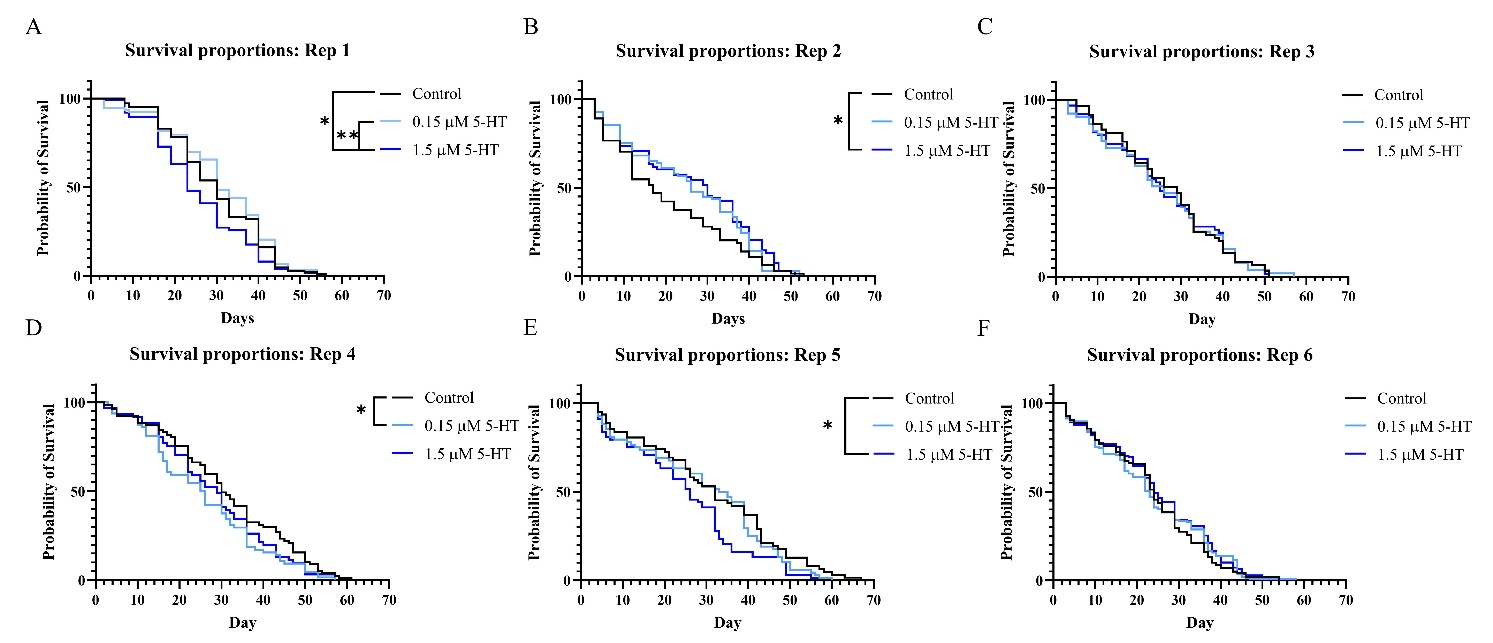


Supplementary Figure 2. Probably of survival of uninfected *A. stephensi* provisioned with weekly blood meals supplemented with 0.15 µM 5-HT, 1.5 µM 5-HT or with an equivalent volume of water (control). Kaplan-Meier analysis of survival with the log-rank test was used to compare groups within each replicate. (A) Replicate 1 (Rep 1), *control vs 1.5 μM p = 0.0147, **0.15 μM vs 1.5 μM p = 0.0015. (B) Replicate 2 (Rep 2), *control vs 1.5 μM p = 0.0448. (C) Replicate 3 (Rep 3), no significance. (D) Replicate 4 (Rep 4), *control vs 0.15 μM p = 0.0309. (E) Replicate 5 (Rep 5), *control vs 1.5 μM p = 0.0175. (F) Replicate 6 (Rep 6), no significance.


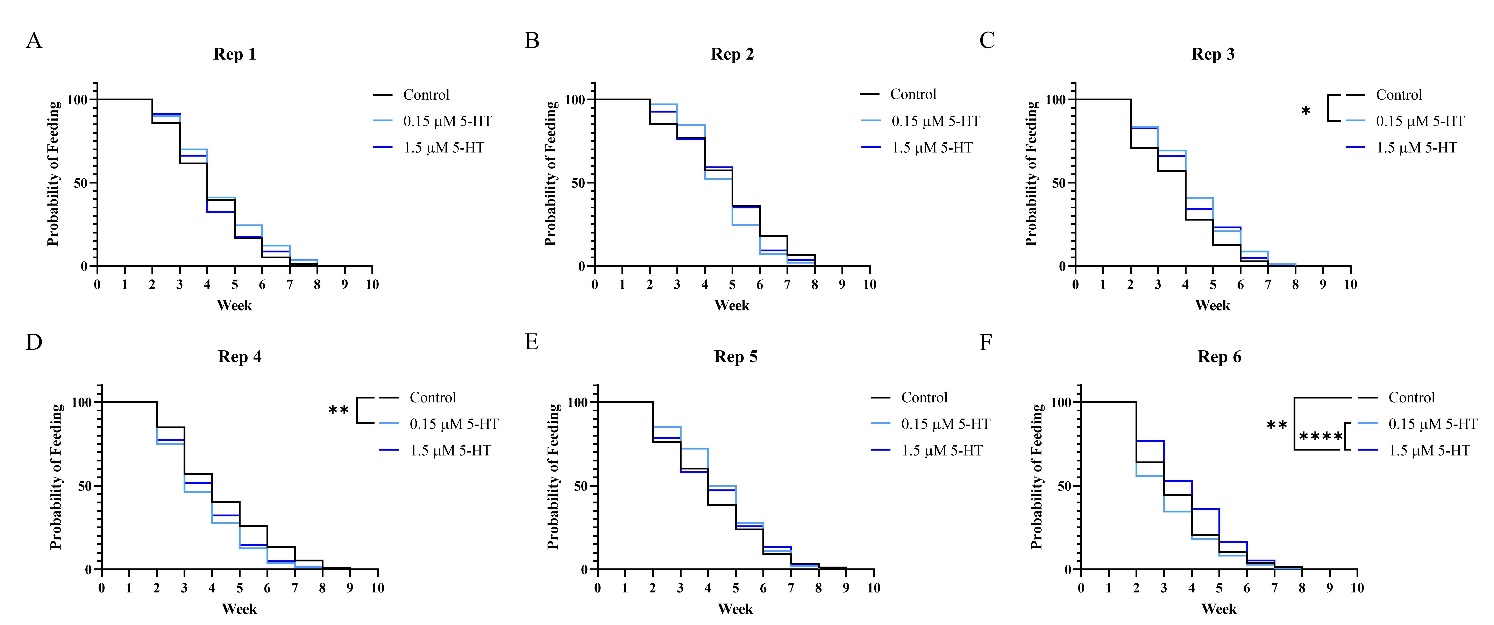


Supplementary Figure 3. Probability of blood feeding over lifespan of uninfected female *A. stephensi* provisioned with weekly blood meals supplemented with 0.15 µM 5-HT, 1.5 µM 5-HT or with an equivalent volume of water (control). Kaplan-Meier analysis of feeding cessation with the log-rank test was used to compare groups within each replicate. (A) Replicate 1 (Rep 1), no significance. (B) Replicate 2 (Rep 2), no significance. (C) Replicate 3 (Rep 3), *control vs 0.15 μM p = 0.0263. (D) Replicate 4 (Rep 4), **control vs 0.15 μM p = 0.0075. (E) Replicate 5 (Rep 5), no significance. (F) Replicate 6 (Rep 6), **control vs 1.5 μM p = 0.0067, ****0.15 μM vs 1.5 μM p < 0.0001.


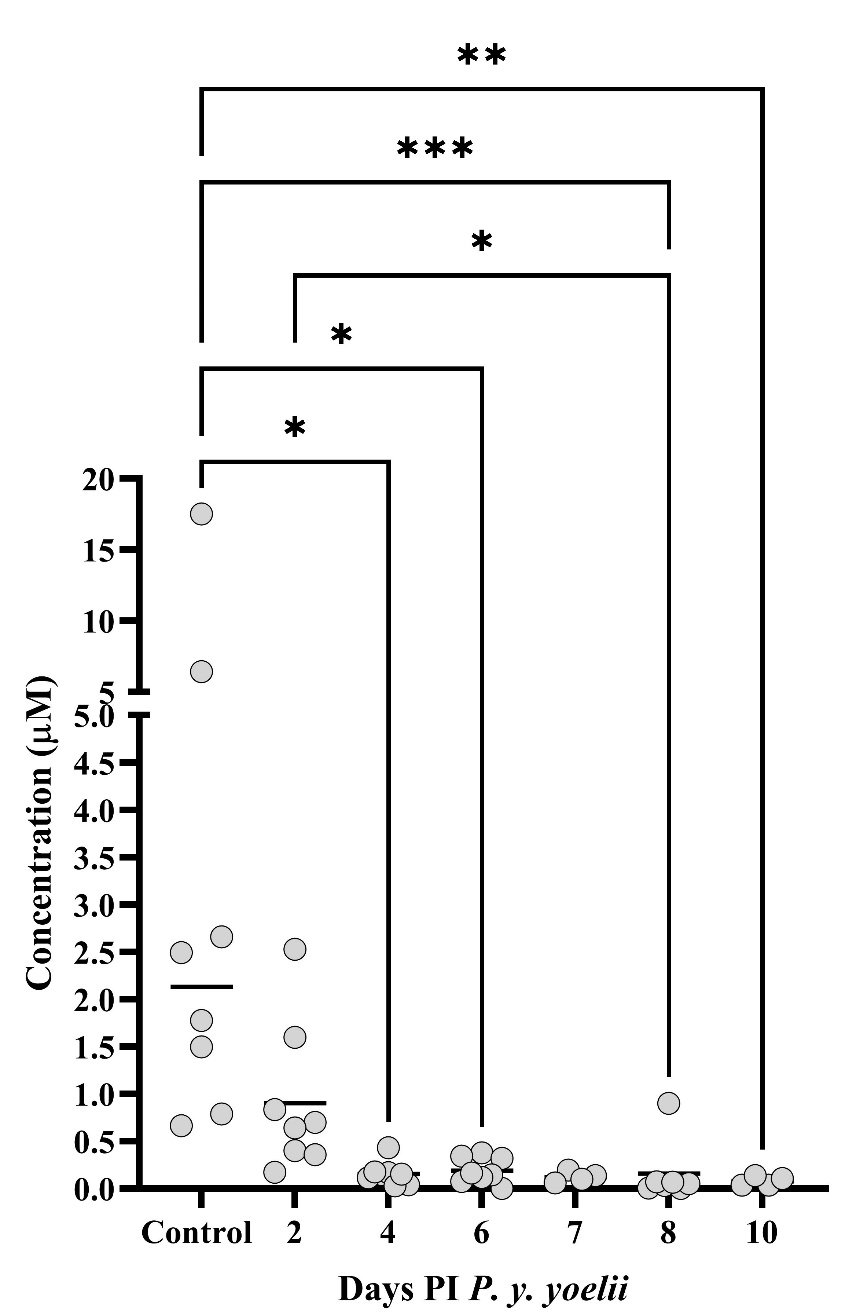


Supplementary Figure 4. Plasma serotonin levels in CD-1 mice infected with *P. yoelii yoelii* 17XNL from 2-10 d post-infection (PI). N = 2; ANOVA (α = 0.05), *control vs 4 d p = 0.0203, *control vs 6 d p = 0.0338, ***control vs 8 d p = 0.0004, **control vs 10 d p = 0.0062, *2 d vs 8 d p = 0.0202.


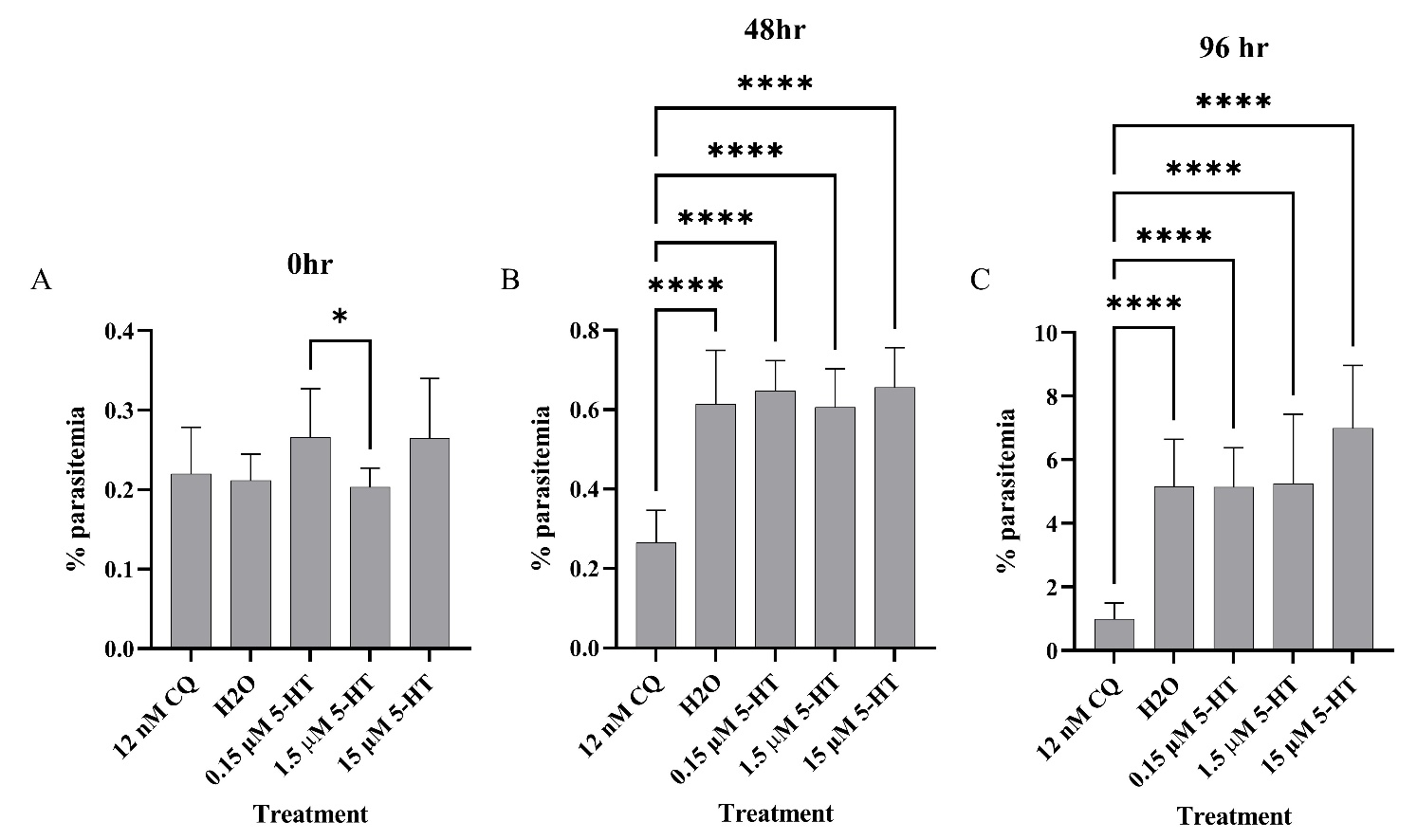


Supplementary Figure 5. Serotonin treatment of *P. falciparum* NF54 *in vitro* had no significant direct effects on parasite growth. Chloroquine (CQ) was used as a positive control for parasite death. (A) Initial parasitemia in all groups prior to treatment. (B) Parasitemia at 48 h (one growth cycle) following treatment with 0.15 µM 5-HT, 1.5 µM 5-HT, and 15 µM 5-HT, an equivalent volume of water used to deliver 5-HT treatments or only media added to parasites. Human RBCs were included to indicate the specificity of flow cytometric detection of fluorescence. (C) Parasitemia at 96 h after treatment (two growth cycles). N = 4 replicates analyzed per treatment, and each analyzed in duplicate; one-way ANOVA (α = 0.05), * p = 0.0470, **** p < 0.0001.

## Supplementary Tables

Supplementary Table 1 A. Impact of ingested 5-HT on *A. stephensi* reproduction. Clutch size of individual *A. stephensi* females during three gonotrophic cycles (GC).

|  |  | Control | | | 0.15 µM 5-HT | | | 1.5 µM 5-HT | | |
| --- | --- | --- | --- | --- | --- | --- | --- | --- | --- | --- |
|  |  | Median | Mean | SE | Median | Mean | SE | Median | Mean | SE |
|  | GC 1 | 51 | 48.91 | 2.053 | 37.5 | 38.28 | 3.522 | 32 | 30.04 | 2.262 |
| Rep 1 | GC 2 | 46 | 42.34 | 3.450 | 33 | 32.92 | 6.118 | 45 | 41.33 | 4.601 |
|  | GC 3 | 41 | 40.17 | 3.066 | 31 | 34.17 | 6.061 | 44 | 40.56 | 3.751 |
|  | GC 1 | 46 | 45.63 | 3.711 | 48 | 44.64 | 3.349 | 43 | 41.65 | 3.406 |
| Rep 2 | GC 2 | 53 | 50.32 | 4.650 | 46 | 42.7 | 4.414 | 46.5 | 39.17 | 4.155 |
|  | GC 3 | 21 | 25.15 | 5.693 | 36 | 33.83 | 6.794 | 42 | 32.9 | 4.829 |
|  | GC 1 | 36 | 30.29 | 5.835 | 28 | 31.74 | 4.776 | 45 | 43.09 | 3.208 |
| Rep 3 | GC 2 | 45 | 41.57 | 3.578 | 43 | 37.89 | 5.550 | 47 | 39 | 5.306 |
|  | GC 3 | 38 | 38.6 | 3.932 | 46 | 43.33 | 5.504 | 38 | 40 | 3.919 |
|  | GC 1 | 38 | 38.31 | 3.448 | 48 | 47.03 | 2.729 | 53.5 | 52.33 | 3.043 |
| Rep 4 | GC 2 | 46.5 | 47.43 | 4.760 | 56 | 53.43 | 4.119 | 59.5 | 56.09 | 3.833 |
|  | GC 3 | 54 | 55.91 | 7.371 | 52 | 52.44 | 4.272 | 61.5 | 56.25 | 7.093 |
|  | GC 1 | 51 | 49.45 | 4.284 | 49.5 | 45.33 | 6.687 | 41 | 35.7 | 5.983 |
| Rep 5 | GC 2 | 69.5 | 67.78 | 6.232 | 60 | 53.86 | 5.669 | 66 | 61.07 | 4.469 |
|  | GC 3 | 51 | 49.67 | 5.921 | 49 | 41 | 7.541 | 70 | 61.09 | 10.79 |

Supplementary Table 1 B. Impact of ingested 5-HT on *A. stephensi* reproduction. The number of mosquitoes that oviposited or did not oviposit during three gonotrophic cycles (GC).

|  |  | Control | | 0.15 µM 5-HT | | 1.5 µM 5-HT | |
| --- | --- | --- | --- | --- | --- | --- | --- |
|  |  | Eggs | No Eggs | Eggs | No Eggs | Eggs | No Eggs |
|  | GC 1 | 32 | 8 | 32 | 8 | 26 | 14 |
| Rep 1 | GC 2 | 35 | 3 | 13 | 4 | 24 | 8 |
|  | GC 3 | 23 | 4 | 12 | 3 | 18 | 3 |
|  | GC 1 | 35 | 5 | 36 | 4 | 37 | 3 |
| Rep 2 | GC 2 | 19 | 2 | 20 | 2 | 30 | 3 |
|  | GC 3 | 13 | 0 | 12 | 0 | 20 | 2 |
|  | GC 1 | 17 | 23 | 27 | 13 | 34 | 6 |
| Rep 3 | GC 2 | 7 | 14 | 19 | 6 | 21 | 1 |
|  | GC 3 | 5 | 6 | 12 | 0 | 9 | 11 |
|  | GC 1 | 26 | 14 | 33 | 7 | 30 | 10 |
| Rep 4 | GC 2 | 14 | 10 | 23 | 3 | 22 | 3 |
|  | GC 3 | 11 | 2 | 9 | 4 | 12 | 3 |
|  | GC 1 | 11 | 29 | 6 | 34 | 10 | 30 |
| Rep 5 | GC 2 | 18 | 7 | 14 | 6 | 15 | 10 |
|  | GC 3 | 9 | 6 | 6 | 8 | 11 | 10 |

Supplementary Table 2 A. Percent survival of uninfected mosquitoes provisioned with 0.15 µM 5-HT, 1.5 µM 5-HT or with an equivalent volume of water diluent (control) in a weekly bloodmeal.

|  | Comparison | Log-Rank (Mantel-Cox) | Significantly different? | Gehan-Breslow-Wilcoxon | Significantly different? |
| --- | --- | --- | --- | --- | --- |
|  | Control – 0.15 µM 5-HT | 0.3581 | N | 0.7993 | N |
| Rep 1 | Control – 1.5 µM 5-HT | 0.0147 | Y | 0.0052 | Y |
|  | 0.15 µM 5-HT – 1.5 µM 5-HT | 0.0015 | Y | 0.0008 | Y |
|  | Control – 0.15 µM 5-HT | 0.1343 | N | 0.0607 | N |
| Rep 2 | Control – 1.5 µM 5-HT | 0.0448 | Y | 0.0314 | Y |
|  | 0.15 µM 5-HT – 1.5 µM 5-HT | 0.5159 | N | 0.6571 | N |
|  | Control – 0.15 µM 5-HT | 0.8906 | N | 0.6075 | N |
| Rep 3 | Control – 1.5 µM 5-HT | 0.8366 | N | 0.7079 | N |
|  | 0.15 µM 5-HT – 1.5 µM 5-HT | 0.9897 | N | 0.8637 | N |
|  | Control – 0.15 µM 5-HT | 0.0309 | Y | 0.0286 | Y |
| Rep 4 | Control – 1.5 µM 5-HT | 0.1546 | N | 0.2405 | N |
|  | 0.15 µM 5-HT – 1.5 µM 5-HT | 0.4306 | N | 0.2961 | N |
|  | Control – 0.15 µM 5-HT | 0.3161 | N | 0.5937 | N |
| Rep 5 | Control – 1.5 µM 5-HT | 0.0175 | Y | 0.0371 | Y |
|  | 0.15 µM 5-HT – 1.5 µM 5-HT | 0.0696 | N | 0.0646 | N |
|  | Control – 0.15 µM 5-HT | 0.5871 | N | 0.8892 | N |
| Rep 6 | Control – 1.5 µM 5-HT | 0.2346 | N | 0.4098 | N |
|  | 0.15 µM 5-HT – 1.5 µM 5-HT | 0.5268 | N | 0.3870 | N |

Supplementary Table 2 B. Median day of survival of uninfected mosquitoes provisioned with 0.15 µM 5-HT, 1.5 µM 5-HT or with an equivalent volume of water (control) in a weekly bloodmeal. N = 6; one-way ANOVA (α = 0.05), no significance.

|  | Control | 0.15 μM 5-HT | 1.5 μM 5-HT |
| --- | --- | --- | --- |
| Rep 1 | 30 | 30 | 23 |
| Rep 2 | 17 | 26 | 30 |
| Rep 3 | 29 | 26 | 25 |
| Rep 4 | 30 | 25.5 | 29 |
| Rep 5 | 32 | 34 | 26 |
| Rep 6 | 24 | 23 | 24 |

Supplementary Table 3 A. Percent blood feeding cessation of uninfected *A. stephensi* females provisioned with 0.15 µM 5-HT, 1.5 µM 5-HT or with an equivalent volume of water diluent (control) in weekly bloodmeals over time.

|  | Comparison | Log-Rank (mantel-cox) | Significantly different? | Gehan-Breslow-Wilcoxon | Significantly different? |
| --- | --- | --- | --- | --- | --- |
|  | Control – 0.15 µM 5-HT | 0.1621 | N | 0.2548 | N |
| Rep 1 | Control – 1.5 µM 5-HT | 0.6824 | N | 0.8281 | N |
|  | 0.15 µM 5-HT – 1.5 µM 5-HT | 0.3756 | N | 0.3525 | N |
|  | Control – 0.15 µM 5-HT | 0.1630 | N | 0.5163 | N |
| Rep 2 | Control – 1.5 µM 5-HT | 0.6106 | N | 0.9027 | N |
|  | 0.15 µM 5-HT – 1.5 µM 5-HT | 0.3987 | N | 0.5894 | N |
|  | Control – 0.15 µM 5-HT | 0.0263 | Y | 0.0291 | Y |
| Rep 3 | Control – 1.5 µM 5-HT | 0.0974 | N | 0.1036 | N |
|  | 0.15 µM 5-HT – 1.5 µM 5-HT | 0.5218 | N | 0.5685 | N |
|  | Control – 0.15 µM 5-HT | 0.0075 | Y | 0.0200 | Y |
| Rep 4 | Control – 1.5 µM 5-HT | 0.0608 | N | 0.1319 | N |
|  | 0.15 µM 5-HT – 1.5 µM 5-HT | 0.5387 | N | 0.5270 | N |
|  | Control – 0.15 µM 5-HT | 0.3899 | N | 0.1702 | N |
| Rep 5 | Control – 1.5 µM 5-HT | 0.6302 | N | 0.6100 | N |
|  | 0.15 µM 5-HT – 1.5 µM 5-HT | 0.7552 | N | 0.4269 | N |
|  | Control – 0.15 µM 5-HT | 0.0837 | N | 0.0532 | N |
| Rep 6 | Control – 1.5 µM 5-HT | 0.0067 | Y | 0.0019 | Y |
|  | 0.15 µM 5-HT – 1.5 µM 5-HT | <0.0001 | Y | <0.0001 | Y |

Supplementary Table 3 B. Median week of blood feeding cessation of uninfected female *A. stephensi* provisioned with 0.15 µM 5-HT, 1.5 µM 5-HT or with an equivalent volume of water diluent (control) in weekly bloodmeals over time. N = 6; one-way ANOVA (α = 0.05), no significance.

|  | Control | 0.15 μM 5-HT | 1.5 μM 5-HT |
| --- | --- | --- | --- |
| Rep 1 | 4 | 4 | 4 |
| Rep 2 | 5 | 5 | 5 |
| Rep 3 | 4 | 4 | 4 |
| Rep 4 | 4 | 3 | 4 |
| Rep 5 | 4 | 4.5 | 4 |
| Rep 6 | 3 | 3 | 4 |

Supplementary Table 4. Significant correlations and their corresponding Pearson p value and r value for uninfected *A. stephensi* variables including gonotrophic cycle (GC), proportion females oviposited, and tendency to take a second blood meal. Significant correlations and their corresponding Pearson p value and r value for *P. y. yoelii 17XNL* infected *A. stephensi* variables including oocyst and sporozoite infection intensities and prevalences and tendency to take a second blood meal. Uninfected mosquitoes, N = 5; Procrustes analysis, *control vs 0.15µM, p = 0.0480, *control vs 1.5µM, p = 0.0229, *0.15µM vs 1.5µM, p = 0.0001. *P. y. yoelii* infected mosquitoes, N = 6; Procrustes analysis. *control vs 1.5µM, p = 0.0198, *0.15µM vs 1.5µM, p = 0.046.

| Parasite | Treatment | Variable 1 | Variable 2 | p value | r value |
| --- | --- | --- | --- | --- | --- |
| Uninfected | Control | GC1 clutch size | GC2 proportion oviposited | 0.0381 | 0.90 |
| Uninfected | Control | GC 1 proportion oviposited | GC3 proportion oviposited | 0.0490 | 0.88 |
| Uninfected | Control | GC2 proportion oviposited | total eggs | 0.0275 | 0.92 |
| Uninfected | Control | total eggs | proportion fed 14 d | 0.0034 | 0.98 |
| Uninfected | Control | median week of feeding cessation | proportion fed 4-7 d | 0.0216 | 0.88 |
| Uninfected | 0.15 µM 5-HT | GC2 proportion oviposited | total eggs | 0.0135 | 0.95 |
| Uninfected | 1.5 µM 5-HT | GC 1 proportion oviposited | GC2 proportion oviposited | 0.0108 | 0.96 |
| Uninfected | 1.5 µM 5-HT | GC3 clutch size | GC2 clutch size | 0.0048 | 0.97 |
| Uninfected | 1.5 µM 5-HT | GC3 proportion oviposited | proportion fed 14 d | 0.0224 | -0.93 |
| *P. y. yoelii* 17XNL | Control | oocysts intensity | sporozoite prevalence | 0.0106 | 0.91 |
| *P. y. yoelii* 17XNL | 0.15 µM 5-HT | proportion fed 4 d | sporozoite intensity | 0.0261 | -0.87 |
| *P. y. yoelii* 17XNL | 1.5 µM 5-HT | proportion fed 4 d | oocyst prevalence | 0.0400 | -0.83 |
| *P. y. yoelii* 17XNL | 1.5 µM 5-HT | proportion fed 4 d | sporozoite prevalence | 0.0115 | -0.91 |
| *P. y. yoelii* 17XNL | 1.5 µM 5-HT | oocysts intensity | oocyst prevalence | 0.0020 | 0.96 |
| *P. y. yoelii* 17XNL | 1.5 µM 5-HT | oocyst prevalence | sporozoite prevalence | 0.0467 | 0.82 |
